# Circulating vitamin C concentration and risk of cancers: a Mendelian randomization study

**DOI:** 10.1186/s12916-021-02041-1

**Published:** 2021-07-30

**Authors:** Yuanqing Fu, Fengzhe Xu, Longda Jiang, Zelei Miao, Xinxiu Liang, Jian Yang, Susanna C. Larsson, Ju-Sheng Zheng

**Affiliations:** 1 Key Laboratory of Growth Regulation and Translational Research of Zhejiang Province, School of Life Sciences, Westlake University, 18 Shilongshan Rd, Cloud Town, Hangzhou, 310024 China; 2 Westlake Intelligent Biomarker Discovery Lab, Westlake Laboratory of Life Sciences and Biomedicine, Hangzhou, China; 3 Institute of Basic Medical Sciences, Westlake Institute for Advanced Study, Hangzhou, China; 4 Institute for Molecular Bioscience, The University of Queensland, Brisbane, QLD Australia; 5 Unit of Cardiovascular and Nutritional Epidemiology, Institute of Environmental Medicine, Karolinska Institute, Stockholm, Sweden; 6 Department of Surgical Sciences, Uppsala University, Uppsala, Sweden

**Keywords:** Circulating vitamin C, Site-specific cancers, Mendelian randomization analysis

## Abstract

**Background:**

Circulating vitamin C concentrations have been associated with several cancers in observational studies, but little is known about the causal direction of the associations. This study aims to explore the potential causal relationship between circulating vitamin C and risk of five most common cancers in Europe.

**Methods:**

We used summary-level data for genetic variants associated with plasma vitamin C in a large vitamin C genome-wide association study (GWAS) meta-analysis on 52,018 Europeans, and the corresponding associations with lung, breast, prostate, colon, and rectal cancer from GWAS consortia including up to 870,984 participants of European ancestry. We performed two-sample, bi-directional Mendelian randomization (MR) analyses using inverse-variance-weighted method as the primary approach, while using 6 additional methods (e.g., MR-Egger, weighted median-based, and mode-based methods) as sensitivity analysis to detect and adjust for pleiotropy. We also conducted a meta-analysis of prospective cohort studies and randomized controlled trials to examine the association of vitamin C intakes with cancer outcomes.

**Results:**

The MR analysis showed no evidence of a causal association of circulating vitamin C concentration with any examined cancer. Although the odds ratio (OR) per one standard deviation increase in genetically predicted circulating vitamin C concentration was 1.34 (95% confidence interval 1.14 to 1.57) for breast cancer in the UK Biobank, this association could not be replicated in the Breast Cancer Association Consortium with an OR of 1.05 (0.94 to 1.17). Smoking initiation, as a positive control for our reverse MR analysis, showed a negative association with circulating vitamin C concentration. However, there was no strong evidence of a causal association of any examined cancer with circulating vitamin C. Sensitivity analysis using 6 different analytical approaches yielded similar results. Moreover, our MR results were consistent with the null findings from the meta-analysis exploring prospective associations of dietary or supplemental vitamin C intakes with cancer risk, except that higher dietary vitamin C intake, but not vitamin C supplement, was associated with a lower risk of lung cancer (risk ratio: 0.84, 95% confidence interval 0.71 to 0.99).

**Conclusions:**

These findings provide no evidence to support that physiological-level circulating vitamin C has a large effect on risk of the five most common cancers in European populations, but we cannot rule out very small effect sizes.

**Supplementary Information:**

The online version contains supplementary material available at 10.1186/s12916-021-02041-1.

## Background

Vitamin C, an essential micronutrient abundant in fruits and vegetables, is essential for many physiological processes in humans [1, 2]. Due to its beneficial effects on redox imbalance, epigenetic reprogramming, oxygen sensing regulation, host immunity, and collagen synthesis, all of which are involved in tumor angiogenesis, treatment evasion, or metastasis, many studies have suggested anticancer potential of vitamin C [2–6]. Prior studies examining the therapeutic effect of vitamin C on cancer found that vitamin C at pharmacological concentrations from intravenous dosing, but not physiological vitamin C from oral dosing, exerted clinical benefits among cancer patients [7–9]. However, whether lifelong exposure to high physiological concentration of vitamin C has a protective effect on cancers is still largely unknown.

Observational studies support an inverse correlation between circulating vitamin C and cancers [10, 11]. However, the possibility of reverse causation could not be ruled out, as cancer-induced oxidative stress and reactive oxygen species formation might increase the consumption of antioxidants including circulating vitamin C, and cancer-related symptoms such as impaired taste, dysphagia, nausea, and vomiting could also contribute to an unbalanced dietary intake of vitamin C. Many prospective cohort studies have examined the associations between dietary or supplementary intake of vitamin C and risk of various types of cancers, but the conclusions were inconsistent [12–17]. In contrast to observational studies, randomized controlled trials (RCT) of vitamin C supplements could potentially help establish the causal relationship. Several RCTs on this topic showed no effect of vitamin C supplementation on the risk of cancers, but the number of incident cases of site-specific cancers was small [13, 18–21]. Therefore, whether the associations between circulating vitamin C and cancers are causal, and the direction of the causal associations (if any) are still unknown. Mendelian randomization (MR) analysis, exploiting inherent properties of common genetic variation for a modifiable environmental exposure of interest, has become a widely used approach to explore the potential causal relations between environmental exposures and diseases [22]. By applying a bi-directional MR approach, on the one hand, we can explore whether circulating vitamin C casually affects the cancer risk, and on the other hand, we can examine whether the genetic predisposition of cancer risk causally influence the circulating vitamin C levels. To date, there has been no MR analysis addressing these questions.

In the present study, we applied a bi-directional MR approach to estimate the putative causal relationships between circulating vitamin C concentrations and risk of site-specific cancers, including lung and bronchus, breast, prostate, colon, and rectal cancers, which together represent half of the overall burden of cancer in Europe [23]. To make comparison with prospective observational or interventional studies, we also conducted a meta-analysis to comprehensively summarize the results of prospective studies assessing the effect of vitamin C intakes on the cancer outcomes.

## Methods

### Putative causal association of circulating vitamin C concentrations with risk of the site-specific cancers

**Fig. 1:**
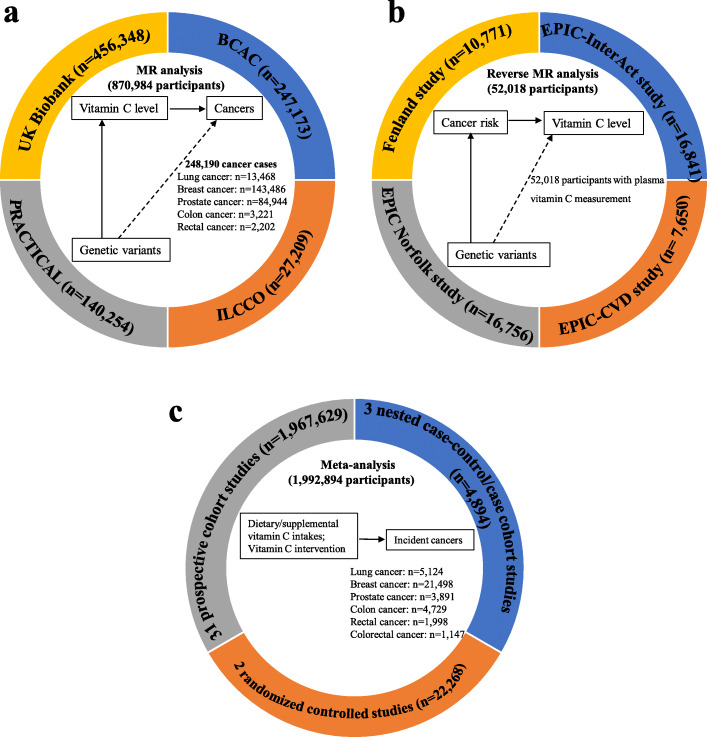
Overview of the design of the present study. **a** Design of the Mendelian randomization analysis of putative causal associations of circulating vitamin C concentrations with risk of the site-specific cancers. 456,348 participants in the UK Biobank, 247,173 participants in the BCAC, 27,209 participants in the ILCCO, and 140,254 participants in the PRACTICAL were included. **b** Design of reverse Mendelian randomization analysis of putative causal associations of site-specific cancer risk with circulating vitamin C concentrations. 10,771 participants in the Fenland study, 16,841 in the EPIC-InterAct study, 7650 participants in the EPIC-CVD study, and 16,756 participants in the EPIC Norfolk study were included. **c** Meta-analysis of prospective studies exploring associations of dietary or supplemental vitamin C intakes with site-specific cancer risk. 31 prospective cohort studies involving 1,967,629 participants, 3 nested case-control studies involving 4894 participants, and 2 RCTs involving 22,268 participants were included. ILCCO, International Lung Cancer Consortium; BCAC, the Breast Cancer Association Consortium; PRACTICAL, the Prostate Cancer Association Group to Investigate Cancer-Associated Alterations in the Genome

Figure 1 provides an overview of the participating studies and design of the present study. The first key component of our study design involved examining causal association of circulating vitamin C concentrations with risk of the site-specific cancers. Genetic instruments (SNPs) of circulating vitamin C concentration were obtained from the up-to-date genome-wide association study (GWAS) [24], which identified 11 plasma vitamin C-associated SNPs explaining 1.87% of the variance of plasma vitamin C. Briefly, this GWAS comprised up to 52,018 individuals of European ancestry from 4 studies, including the Fenland study, the European Prospective Investigation into Cancer and Nutrition (EPIC)-InterAct study, the EPIC Norfolk study, and the EPIC-CVD study. The SNPs include a known locus at *SLC23A1*(rs33972313) and 10 novel genetic loci ([*RER1*]-rs6693447, [*SLC23A3*]-rs13028225, [*RGS14*]-rs10051765, [*GSTA5*]-rs7740812, [*FADS1*]-rs174547, [*SNRPF*]-rs117885456, [*CHPT1*]-rs2559850, [*AKT1*]-rs10136000, [*MAF*]-rs56738967, and [*BCAS3*]-rs9895661).

Summary-level data for the association between genetic variants and 5 site-specific cancer outcomes (i.e., lung (including bronchus), prostate, breast, colon, and rectal cancer) were retrieved by running the GWAS for each cancer in the UK Biobank using the newly developed fastGWA-glmm tool [25]. The UK Biobank is a cohort study of about half million adults (40–69 years of age at baseline) recruited between 2006 and 2010 [26]. In the current analyses, we included 456,348 UK Biobank participants.

To replicate our findings on the associations between the vitamin C-related SNPs and cancers in the UK Biobank dataset, publicly available summary-level data for lung, prostate, and breast cancer were obtained from the International Lung Cancer Consortium (ILCCO), the Prostate Cancer Association Group to Investigate Cancer Associated Alterations in the Genome (PRACTICAL) consortium, and the Breast Cancer Association Consortium (BCAC), respectively [27–30]. Briefly, the ILCCO was established in 2004, with the goal of sharing compatible data from lung cancer epidemiology studies around the world to maximize statistical power. We acquired summary data from the ILCCO using the MR-Base database, involving 27,209 participants of European ancestry (15,861 controls, and 11,348 cases including 3442 lung adenocarcinoma and 3275 lung squamous cell carcinoma) [27, 28]. The PRACTICAL consortium and BCAC aimed to identify genes that were related to the risk of prostate and breast cancer, respectively, by combining data from many studies. We retrieved publicly available summary-level data of 140,254 participants of European ancestry (79,148 prostate cancer cases and 61,106 controls) from a meta-analysis of 8 GWASs included in the PRACTICAL consortium [29]. The summary results from another meta-analysis of the BCAC and 11 other breast cancer GWASs involving 247,173 participants of European ancestry (133,384 breast cancer cases and 113,789 controls) were also included in the current analysis [30, 31]. Thus, a total of 248,111 cancer cases and up to 644,984 controls were included for exploring the potential effects of circulating vitamin C on cancer risk.

### Putative causal associations of a site-specific cancer risk with circulating vitamin C concentrations

Secondly, we performed a reverse MR to examine causal associations of site-specific cancer risk with circulating vitamin C concentrations. Genetic instruments of lung cancer, prostate cancer, breast cancer, colorectal cancer, and smoking initiation were obtained from the most up-to-date GWASs. Significant SNPs (n=7) for lung cancer were reported by McKay et al. [32]. The SNPs (n=147) for prostate cancer were obtained from the PRACTICAL consortium [29]. For breast cancer, we used 32 SNPs reported by Zhang et al. and 178 SNPs summarized by Ahearn et al. as instrumental variables [30, 33]. For colorectal cancer, we obtained significant SNPs (n=79) provided by Law et al. [34]. The independent SNPs (n=129) for smoking initiation at the genome-wide level (p<5×10^-8^) were identified from a GWAS study reported by Liu et al. [35]. Summary-level data for the association between genetic variants and circulating vitamin C concentration were retrieved from the GWAS summary statistics of a recent GWAS of up to 52,018 individuals [24].

### Selection of genetic variants

The genetic variants used as instrumental variables for the exposure in MR analyses should be uncorrelated, as assumed in most MR methods, and strongly associated (*p* <5×10^-8^) with the exposure of interest. For the SNPs that were not reported in the GWAS summary statistics for outcomes, we used the proxy in phase (i.e., both are located on the maternal or paternal chromosome) and in high linkage disequilibrium (LD) (r^2^ >0.8) with the original SNPs and discarded the SNPs when no proxy was available (Additional file 1: Supplemental Table 1& Additional file 2: Supplemental Table 2). The LD calculation was based on 503 European samples from the 1000 Genomes phase 3 data [36]. We selected 10 out of the 11 plasma vitamin C-related SNPs as genetic instruments for analysis, as a previous GWAS study had reported pleiotropic effects of the variant (rs174547) in the *FADS1* gene, which was associated with a large number of glycerophospholipids and sphingolipids [24]. As tested in the GWAS study, the selected 10 SNPs could be assumed as satisfying the instrumental variable assumptions 1 and 2 (i.e., the genetic variants are strongly associated with circulating vitamin C concentration, independent of any potential non-genetic confounders) [24]. For the cancer-related SNPs that were not reported in the GWAS summary statistics for outcomes (i.e., circulating vitamin C), we instead used the available proxy and discarded the SNPs when no proxy was available. Thereafter, we further checked whether the SNPs were associated with cancer susceptibility at genome-wide significance level (p<5*10^-8^) and excluded those were not. We summarized the included SNPs in the Supplemental Table 2 (Additional file 2).

### Comparison with prospective observational or interventional studies

We conducted a meta-analysis of previously published prospective cohort studies or randomized controlled trials involving 1,992,894 participants, to provide a comprehensive comparison with our MR findings. In the Additional file 3 (Table S1-S2 & Figure S1-S8), we described methods and results of the meta-analysis in detail. The protocol for the meta-analysis was published in the PROSPERO database (www.crd.york.ac.uk/PROSPERO; registration number: CRD42020220405).

### Statistical analysis

Based on the publicly available GWAS summary statistics for vitamin C as well as the cancer outcomes, genetic correlations were estimated through LD score regression, using LDSC v1.0.1 [37, 38]. The minimum detectable odds ratio (OR) per 1 standard deviation (SD) increase in plasma vitamin C concentration was calculated using mRnd online, assuming 80% power at 5% significance level. We then performed a two-sample MR analysis, using effect estimates of “SNP to vitamin C” associations and “SNP to cancer” associations, to investigate causal associations of circulating vitamin C with cancer risks. The results were presented as ORs and 95% confidence intervals (CI) for site-specific cancers per 1-SD increase in circulating vitamin C concentration (ranging from 17.6 to 21.5 μmol/L depending on study populations). To replicate our findings, we repeated the two-sample MR analyses using summary-level data from the ILCCO consortium for lung cancer, from the PRACTICAL consortium for prostate cancer, and from the BCAC consortium for breast cancer.

To estimate the potential causal association of cancer risk with circulating vitamin C concentrations, we performed a reverse MR analysis. As smoking could decrease vitamin C concentration according to prior biological knowledge and studies [39, 40], we examined the causal association of smoking initiation with circulating vitamin C as a positive control for our reverse MR analysis. The results were presented as the SD change in the vitamin C concentrations and 95% confidence intervals per 1-unit change in log of relative risks of site-specific cancers and smoking initiation. Of note, one SNP (rs55781567) in the genetic instrument of lung cancer was located at 5′ untranslated region of *CHRNA5* gene that had been reported to be associated with nicotine addiction, and rs55781567 has been identified as an eQTL for *CHRNA5* [41]. Therefore, we did another MR for lung cancer by excluding this SNP (due to its potential pleiotropic effect on smoking status). Furthermore, we also performed multivariate MR analysis, with adjustment for smoking, to explore causal association between circulating lung cancer and circulating vitamin C.

The principal analyses were conducted using the random-effects inverse-variance-weighted (IVW) approach, assuming that all SNPs are valid instrumental variables [42]. To evaluate the potential violation of the MR assumption 3 (i.e., the genetic variant is associated with outcomes only through their effect on exposures), we applied the following approaches in sensitivity analyses: (1) MR-Egger regression method, which can detected and adjusted for directional pleiotropy [43]; (2) Mode-based estimation (MBE), which has a natural robustness to variants with outlying ratio estimates, and so are not as affected by the presence of a small number of pleiotropic variants as the IVW and MR-Egger methods [44]; (3) weighted median method, which provides a causal estimate if at least 50% of the weight in the analysis comes from valid instrumental variables [45]; (4) MR-pleiotropy residual sum and outlier (MR-PRESSO) method, which can detect and adjust for horizontal pleiotropy [46]; (5) MR-Robust approach that can remove or down-weight the outliers, if the horizontal pleiotropy was present (MR-PRESSO global test: p<0.01) [47]; and (6) MR-Robust adjusted profile score (MR-RAPS) with Huber loss function which can model a random-effects distribution of the pleiotropic effects of genetic variants [48]. For both directions of the MR analysis, we used Cochran’s *Q* statistics to examine the heterogeneity between the SNP-specific estimates and highlighted the weighted median results if significant heterogeneity of the causal associations among different genetic variants was observed.

Unless otherwise specified, all analyses were performed in R, version 3.5.3, and Stata 15.0 (Stata Corp). All p values were 2-sided and associations were considered statistically significant at p<0.05.

## Results

### Characteristics of the selected SNPs and the cancer outcomes

The associations of the 10 selected SNPs with plasma vitamin C concentration were shown in Supplemental Table 3 (Additional file 1). The sample sizes for each site-specific cancer in each participating study were listed in Table 1. This MR study had relatively high power to detect effect sizes of small to moderate magnitude in the replication datasets, while the power was adequate to detect only large effect sizes for most of the cancer outcomes in the UK Biobank dataset (Table 1). For the reverse MR analysis, the characteristics of the selected SNPs associated with site-specific cancer outcomes were summarized in Supplemental Table 2 (Additional file 2).

**Table 1:** Number of cancer cases and controls and statistical power in Mendelian randomization study on association of circulating vitamin C concentration with risk of site-specific cancers

Cancer type	Study/Consortium	Cases	Controls	Minimum detectable OR (R^2^=0.0187)
Lung (bronchus) cancer	UK Biobank	2,120	454,228	0.55/1.45
Prostate cancer		5,796	203,012	0.75/1.25
Breast cancer		10,892	236,648	0.80/1.21
Colon cancer		3,221	453,127	0.64/1.36
Rectal cancer		2,202	454,146	0.56/1.44
**Lung cancer**	ILCCO			
Overall		11,348	15,861	0.77/1.28
Adenocarcinoma		3,442	14,894	0.64/1.42
Squamous cell carcinoma		3,275	15,038	0.63/1.42
**Prostate cancer**	PRACTICAL			
Overall		79,148	61,106	0.89/1.12
**Breast cancer**	BCAC			
Overall		133,384	113,789	0.92/1.09
ER-positive		69,501	105,974	0.90/1.11
ER-negative		21,468	105,974	0.85/1.16

*ILCCO* International Lung Cancer Consortium, *PRACTICAL* the Prostate Cancer Association Group to Investigate Cancer Associated Alterations in the Genome, *BCAC* the Breast Cancer Association Consortium, *OCAC* the Ovarian Cancer Association Consortium

### Bi-directional association of circulating vitamin C and site-specific cancers in MR analysis

**Fig. 2:**
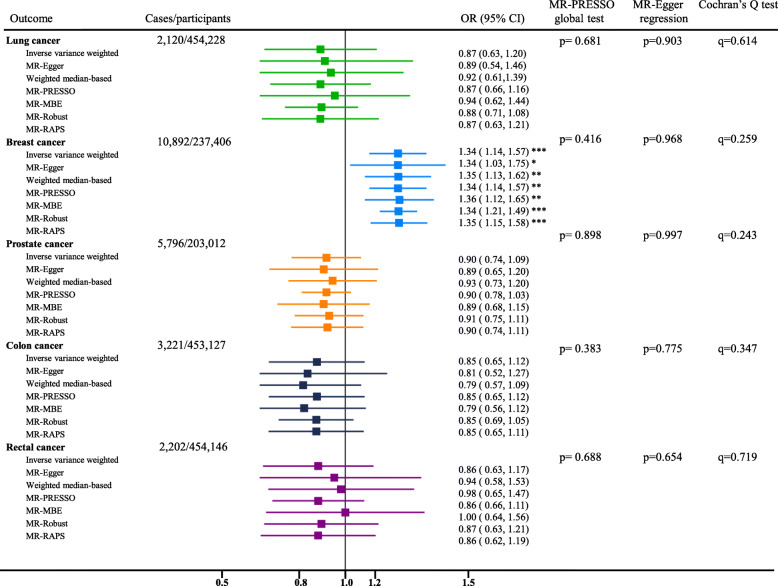
Odds ratios for associations between genetically predicted circulating vitamin C and site-specific cancers in the UK Biobank. The ORs represent the odds ratios per 1-standardized unit (in SD unit) increase in the plasma vitamin C concentration. The random-effects inverse-variance-weighted method was used as the primary approach, while other methods including MR-Egger, weighted median-based, MR-PRESSO, mode-based, MR-Robust, and MR-RAPS were used as sensitivity analyses. The MR-PRESSO global test and MR-Egger regression were used to detect the pleiotropic effects. Using the MR-Egger regression method, the effect of genetic instruments on the exposure is plotted against its effect on the outcome, and an intercept distinct from the origin provides evidence for pleiotropic effects (MR-Egger regression test: p<0.01). We highlight the outlier-corrected MR estimates using MR-PRESSO and MR-Robust if the horizontal pleiotropy was present (MR-PRESSO global test: p<0.01). The q values derived from the Cochran’s *Q* statistics were used to reflect heterogeneity between the SNP-specific estimates, and the weighted median-based results should be highlighted if significant heterogeneity was observed. *Indicates p<0.05, **indicates p<0.01, and ***indicates p<0.001. PRESSO, Pleiotropy Residual Sum and Outlier; MBE, Mode Based Estimation; RAPS, Robust Adjusted Profile Score

The genetic correlation analysis showed that lung cancer, but not other cancers, had significant genetic correlation (r_g_=−0.43, p=0.01) with circulating vitamin C (Additional file 1: Supplemental Table 4). The genetic correlation between smoking initiation and circulating vitamin C was significant (r_g_=−0.24, p<0.0001; Additional file 1: Supplemental Table 4). In MR analysis based on the UK Biobank, genetic predisposition to a higher circulating vitamin C concentration (per 1-SD increment) was not associated with risk of lung and bronchus cancer (OR 0.87; 95% CI 0.63–1.20; p=0.39), prostate cancer (OR 0.90; 95% CI 0.74–1.09, p=0.29), colon cancer (OR 0.85; 95% CI 0.65–1.12; p=0.25), or rectal cancer (OR 0.86; 95% CI 0.63–1.17; p=0.34), but associated with higher odds of breast cancer (OR 1.34; 95% CI 1.14–1.57, p<0.001) (Fig. 2). The forest and scatter plots for each SNP-CA association and the results of heterogeneity test were summarized in the Additional file 4 (Figure S1-S8).

**Fig. 3:**
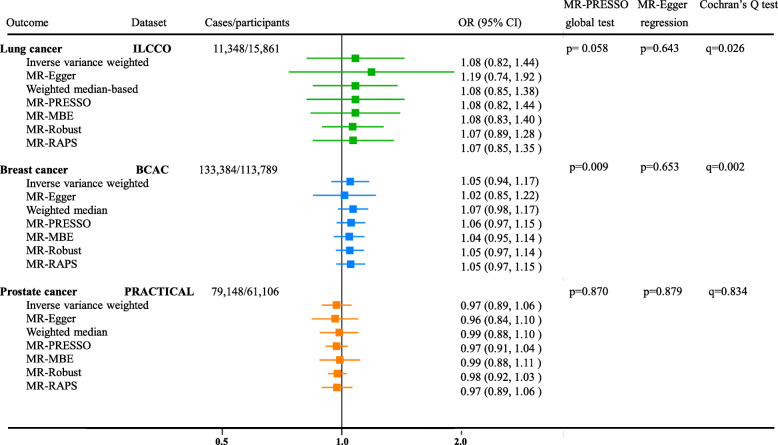
Odds ratios for the associations between genetically predicted circulating vitamin C and site-specific cancers in the replication datasets. The ORs represent the odds ratios per 1-standardized unit (in SD unit) increase in the genetically predicted plasma vitamin C concentration. The random-effects inverse-variance-weighted method was used as the primary approach, while other methods including MR-Egger, weighted median-based, MR-PRESSO, mode-based, MR-Robust, and MR-RAPS were used as sensitivity analyses. The MR-PRESSO global test and MR-Egger regression were used to detect the pleiotropic effects. Using the MR-Egger regression method, the effect of genetic instruments on the exposure is plotted against its effect on the outcome, and an intercept distinct from the origin provides evidence for pleiotropic effects (MR-Egger regression test: p<0.01). We highlighted the outlier-corrected MR estimates using MR-PRESSO and MR-Robust if the horizontal pleiotropy was present (MR-PRESSO global test: p<0.01). The q values derived from the Cochran’s *Q* statistics were used to reflect heterogeneity between the SNP-specific estimates, and the weighted median-based results should be highlighted if significant heterogeneity was observed. PRESSO, Pleiotropy Residual Sum and Outlier; MBE, mode-based estimation; RAPS, robust-adjusted profile score; ILCCO, International Lung Cancer Consortium; BCAC, the Breast Cancer Association Consortium; PRACTICAL, the Prostate Cancer Association Group to Investigate Cancer Associated Alterations in the Genome

In the analysis of replication datasets from ILCCO and PRACTICAL consortium, consistently null MR results were observed with an OR of 1.08 (95% CI 0.82–1.44, p=0.58) for lung cancer and an OR of 0.97 (95% CI 0.89–1.06, p=0.50) for prostate cancer (Fig. 3). Moreover, the positive association between genetically predicated circulating vitamin C and breast cancer observed in the UK Biobank could not be replicated in the dataset of BCAC (OR 1.05; 95% CI 0.94–1.17, p=0.38), which included a much larger number of breast cancer cases (Fig. 3). Further random-effect meta-analysis combining the ORs for breast cancer from UK Biobank and BCAC still yielded a null result (OR 1.18; 95% CI 0.93–1.49). Although Cochran’s *Q* test showed significant heterogeneity for the associations of circulating vitamin C with lung cancer and breast cancer, the weighted-median based sensitivity analysis showed consistent results with the primary IVW approach. No significant association of genetically predicted circulating vitamin C concentrations with any subtypes of lung cancer or breast cancer was observed, but the precision of the estimates was relatively low due to small number of cases (Additional file 1: Supplemental Table 5).

**Fig. 4:**
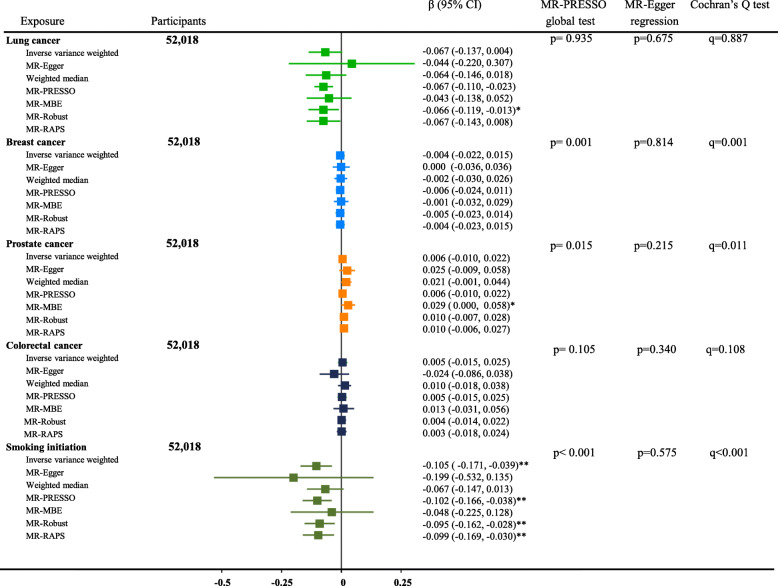
Reverse MR analysis on associations of genetically predicted cancer risk with circulating vitamin C concentration. The β represents the change (in SD unit) in plasma vitamin C concentration per 1-unit increase in genetically predicted cancer risk (logOR). The random-effects inverse-variance-weighted method was used as the primary approach, while other methods including MR-Egger, weighted median-based, MR-PRESSO, mode-based, MR-Robust, and MR-RAPS were used as sensitivity analyses. The MR-PRESSO global test and MR-Egger regression were used to detect the pleiotropic effects. Using the MR-Egger regression method, the effect of genetic instruments on the exposure is plotted against its effect on the outcome, and an intercept distinct from the origin provides evidence for pleiotropic effects (MR-Egger regression test: p<0.01). We highlight the outlier-corrected MR estimates using MR-PRESSO and MR-Robust if the horizontal pleiotropy was present (MR-PRESSO global test: p<0.01). The q values derived from the Cochran’s *Q* statistics were used to reflect heterogeneity between the SNP-specific estimates, and the weighted median-based results should be highlighted if significant heterogeneity was observed. *Indicates p<0.05, **indicates p<0.01. PRESSO, Pleiotropy Residual Sum and Outlier; MBE, mode-based estimation; RAPS, robust-adjusted profile score

We subsequently performed a reverse MR analysis and found that smoking initiation was causally associated with lower concentrations of circulating vitamin C (β=−0.105, 95% CI −0.171 to −0.039, p<0.01). Notably, Cochran’s Q test indicated significant heterogeneity of the causal associations among different genetic variants, and the weighted median-based result showed non-significant association (β=−0.067, 95% CI −0.147 to 0.013, p=0.10). Moreover, our reverse MR analysis found evidences that increased risk of lung cancer was associated with lower concentrations of circulating vitamin C (β=−0.066, 95% CI −0.106 to −0.025, p=0.001), but the association became non-significant after removing the SNP (rs55781567) with potential pleiotropic effect (β=−0.067, 95% CI −0.137 to 0.004, p=0.07) or adjusting for smoking (β=−0.015, 95% CI −0.034 to 0.004, p=0.111; Additional file 1: Supplemental Table 6). No evidence was found to support a causal association of any other tested cancers with circulating vitamin C concentration (Fig. 4).

### Sensitivity analyses of MR

In the MR analysis of associations of genetically predicted circulating vitamin C with site-specific cancers, the MR-PRESSO global test suggested horizontal pleiotropy for the associations of the vitamin C-related genetic variants with breast cancer, while the MR-Egger regression did not indicate any horizontal pleiotropy. In the reverse MR analysis, potential horizontal pleiotropy was only suggested by MR-PRESSO global test for breast cancer- and smoking initiation-related genetic variants. Nevertheless, the significance of the MR estimates remained unchanged after adjustment for the pleiotropy using MR-PRESSO approach. Moreover, compared to the primary IVW method, sensitivity analysis using different MR methods did not substantially change the MR results (Figs. 2, 3, and 4), except that there were several MR approaches yielding significant associations of lung cancer with vitamin C concentration (Fig. 4).

### Observational association between vitamin C intake and risk of cancers

**Fig. 5:**
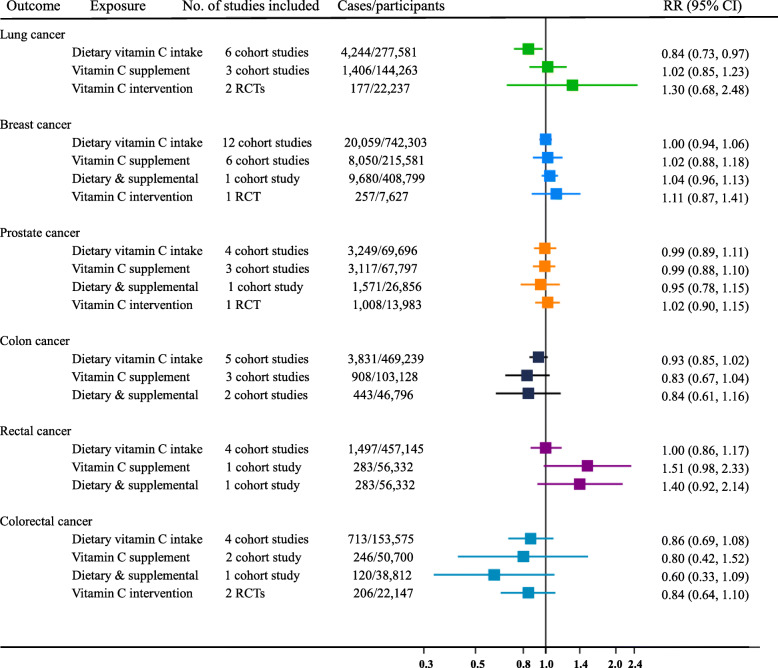
Meta-analysis of the prospective associations between vitamin C intakes with incident site-specific cancers. The summary RRs were pooled by using a random-effects model for the highest versus the lowest category of exposure. The meta-analysis was performed independently for different assessments of exposure (i.e., dietary vitamin C intake, supplemental vitamin C intake, and total vitamin C intake)

By conducting a systematic review, we identified 34 published prospective cohort studies and 2 RCTs with up to 1,992,894 participants (see Additional file 3: Table S2 [12–15, 17, 21, 49–78]). The meta-analysis showed that only dietary vitamin C intakes had a protective effect on lung cancer, with a summary RR of 0.84 (95% CI 0.73 to 0.97), comparing the highest versus the lowest category of exposure. Interestingly, the summary RR of lung cancer for supplemental vitamin C intake was 1.02 (95% CI 0.85 to 1.23) based on cohort studies and 1.30 (95 CI 0.68 to 2.48) based on RCTs, showing no evidence to suggest use of vitamin C supplements. Additionally, consistent null results were observed for any other cancer outcomes, regardless of the sources of vitamin C intake and study designs (Fig. 5; Additional file 3:Fig. S2-S7).

## Discussion

This bi-directional MR analysis based on large-scale genetic consortia provided no evidence to support a causal association of circulating vitamin C concentrations with risk of cancer of the lung and bronchus, prostate, breast, colon, or rectum. Moreover, the meta-analysis of prospective studies of the associations of dietary or supplemental vitamin C intakes with cancer risk did not support the use of vitamin C supplements for prevention of the five cancers.

To the best of our knowledge, this was the first MR analysis to examine the potential bi-directional relationships between circulating vitamin C concentrations and site-specific cancer risk. Previous studies have explored the causal association of circulating vitamin C concentrations instrumented by only one genetic variant (rs33972313) with several health outcomes, including hyperuricaemia, ischemic heart disease, and Alzheimer disease, but not any cancer [79–81].

The association of circulating vitamin C with cancer risk has been examined in several observational studies, most of which focused on total cancer [82–84]. The results of meta-analyses including 5 studies involving 45,758 participants showed that each 50 μmol/L increase in vitamin C concentration was associated with a 26% lower risk of total cancer [85]. Focusing on site-specific cancers, another systematic review reported a significant association of higher plasma vitamin C concentration with lower risk of breast cancer based on case-control studies [86]. One explanation was that the cancer-induced oxidative stress and ROS formation may increase the consumption of vitamin C that acted as an antioxidant; thus, the observed associations in cross-sectional studies may be because of the reverse causality [87].

Interestingly, our reverse MR analysis based on the primary approach (i.e., IVW method) found clues that smoking initiation might causally decrease circulating vitamin C or even mediate the association of lung cancer with circulating vitamin C. However, significant heterogeneity of the causal associations among different genetic variants was observed, and the median-based estimator showed non-significant associations. The sensitivity to the inclusion of invalid IVs may help explain the discrepancies. Specifically, the IVW estimate assumed that all genetic variants are valid instrumental variables, while the weighted median estimate can provide a consistent estimated of the causal effect when up to (not including) 50% of genetic variants are invalid [45]. Therefore, the weighted median estimator is more conservative than IVW approach, especially when heterogeneity between the SNP-specific estimates presents. Nevertheless, other sensitivity analysis methods which are also robust to some violation of the instrumental variable assumptions (e.g., MR-PRESSO, MR-Robust, and MR-RAPS) still yield positive results for inferring causal effects of smoking initiation on circulating vitamin C. Notably, in the present study, smoking initiation served as a positive control for our reverse MR analysis, as previous observational studies showed smoking was associated with decreased vitamin C concentrations (39, 40). The facts that many of our analysis approaches successfully found clues of the association between smoking and circulating vitamin C may validate that our reverse MR approaches would detect signals of causal effects of cancers on plasma vitamin C, if the effect sizes were comparable with that of smoking.

As the circulating vitamin C was rarely measured in prospective cohort studies, most observational studies examined the preventative effects of vitamin C supplementation or dietary vitamin C against cancers [88]. In a meta-analysis of 21 case-control and cohort studies, including 8938 lung cancer cases, the risk of lung cancer decreased by 7% for every 100 mg/day increase in vitamin C intake among men [89]. However, another pooled analysis of women from five prospective studies in the UK Dietary Cohort Consortium did not find evidence of a significant association between vitamin C intakes and breast cancer incidence [90]. Observational studies also yielded controversial results for colorectal cancer. A pooled analysis of prospective cohort studies found that high (>600 mg/day) versus low (≤100mg/day) vitamin C intake was associated with a 19% lower risk of colon cancer [91], but no significant association was observed between vitamin C supplement use and colon cancer risk in a meta-analysis based on three studies conducted in Europe and the USA [92].

In the present study, we performed a more comprehensive and up-to-date meta-analysis of prospective cohort studies and RCTs, involving up to 1,992,894 participants to summarize the potential effect of vitamin C intake on several common site-specific cancers. Our findings also support the abovementioned beneficial association of dietary vitamin C with lung cancer and null findings for breast cancer or colon cancer. However, only dietary vitamin C but not supplemental vitamin C intake exhibit potential protective association with lung cancer. Thus, compared with cross-sectional observational studies, prospective studies, and RCT studies tend to yield more consistent results with our MR findings. Given the null associations discovered in our MR analysis, the abovementioned controversial results based on observational studies raised concern about confounding, as the main sources of dietary vitamin C are fruits and vegetables which are also rich in polyphenols and fibers. Thus, circulating vitamin C might be just a biomarker of fruit and vegetable consumption [24, 93]. Moreover, participants consuming high amounts of fruits and vegetables might be more health conscious.

Over the past decades, although a lot of studies supported the role of vitamin C in cancer prevention, the direction and magnitude of the association are uncertain and contradictory across observational studies [88]. At present, in the context where pharmacological high dose of intravenous vitamin C alone or in combinations with clinically used drugs showed promising efficacy on treating several types of cancers, it is of great public health importance to clarify whether keeping high physiological circulating vitamin C levels through vitamin C intake has a beneficial effect on cancer prevention. The current study did not support a causal association of circulating vitamin C at physiological level with risk of five most common cancers in Europe. As circulating vitamin C cannot be synthesized by humans, and has to be obtained from diet [93], our findings also imply that vitamin C supplementation is unlikely to be helpful for the prevention of the five most common cancers. Of note, our findings do not rule out the potential beneficial effects of fruits and vegetables, which besides vitamin C are rich in numerous phytochemicals and dietary fibers.

Our study has several strengths. First, in addition to the [*SLC23A1*]-rs33972313, which had long been used as the genetic instrument of circulating vitamin C, we further included another 9 up-to-date genetic variants identified in European populations to construct the genetic instrument. Second, our study is the first MR analysis on the relationship between circulating vitamin C and site-specific cancers, based on various large-scale cancer consortium data and the UK Biobank in European populations. The large sample size provides us with enough power to estimate the causal relationship between circulating vitamin C and site-specific cancers. Third, we summarized evidence from published prospective studies for vitamin C intake and incident site-specific cancers, which provides a comprehensive comparison with our MR findings.

This study has several limitations. First, due to limited available datasets for colorectal cancer and other different cancer subtypes, we cannot independently replicate the UK Biobank-derived findings on the colorectal cancer or explore the bi-directional relationships between circulating vitamin C and subtypes of different site-specific cancers, while different cancer subtypes may imply different etiology and pathogenesis. Second, this study can only investigate the potential effects of circulating vitamin C at physiological level on cancer prevention, but not the vitamin C exposure at a pharmaceutical level. Third, despite including data from very large genetic epidemiology networks, our study is not powered to detect very small effects. Lastly, our results are mainly based on participants of European ancestry and may not be generalizable to other ethnic populations.

## Conclusions

The present study did not find evidence to support that high circulating vitamin C concentration at physiological level has a large protective effect on the five most common cancers in European populations. The reported associations between dietary vitamin C and cancer risk in observational studies might be confounded by other components of vitamin C-rich foods.

## Supplementary Information

**Additional file 1: Supplemental Table 1.** The plasma vitamin C-related genetic variants used for the MR analyses. **Supplemental Table 3.** Characteristics of the genetic variants that were used as the instrumental variables for plasma vitamin C concentration. **Supplemental Table 4**. Genetic correlation between vitamin C and site-specific cancers, estimated through linkage disequilibrium score regression. **Supplemental Table 5**. Mendelian randomization estimates of the association between genetically predicted plasma vitamin C concentration and risk of secondary cancer outcomes based on cancer subtypes. **Supplemental Table 6**. Multivariate MR analysis exploring causal association between circulating Vitamin C and lung cancer with adjustment for smoking.

**Additional file 2: Supplemental Table 2a.** Selection of cancer-related genetic variants used for the reverse MR analyses. **Supplemental Table 2b.** Selected instrumental variables or their proxies for the reverse MR analyses.

**Additional file 3: Table S1-S2 & Figure S1-S8.** Supplemental methods and results for the systematic review and meta-analysis. **TableS1.** PubMed search strategy. **TableS2.** Characteristics of the included prospective studies. **FigS1.** Flow diagrams of the literature research. **FigS2.** The association of dietary and supplemental vitamin C intakes with incident lung cancer. **FigS3.** The association of dietary, supplemental and total vitamin C intakes with incident breast cancer. **FigS4.** The association of dietary, supplemental and total vitamin C intakes with incident prostate cancer. **FigS5.** The association of dietary vitamin C intakes with incident colorectal cancer. **FigS6.** The association of supplemental vitamin C intakes with incident colorectal cancer. **FigS7.** The association of total vitamin C intakes with incident colorectal cancer. **FigS8.** Funnel plot for associations of vitamin C intakes with cancers, with Egger’s test adopted to examine the publication bias.

**Additional file 4: Figure S1-S8.** The forest and scatter plots for each SNP-CA association and the results of heterogeneity test. **FigS1.** Genetically predicted associations of plasma vitamin C with lung cancer in the UK biobank dataset. **FigS2.** Genetically predicted associations of plasma vitamin C with breast cancer in the UK biobank dataset. **FigS3.** Genetically predicted associations of plasma vitamin C with prostate cancer in the UK biobank dataset. **FigS4.** Genetically predicted associations of plasma vitamin C with colon cancer in the UK biobank dataset. **FigS5.** Genetically predicted associations of plasma vitamin C with rectal cancer in the UK biobank dataset. **FigS6.** Genetically predicted associations of plasma vitamin C with lung cancer in the dataset from International Lung Cancer Consortium (ILCCO). **FigS7.** Genetically predicted associations of plasma vitamin C with breast cancer in the dataset from the Breast Cancer Association Consortium (BCAC). **FigS8.** Genetically predicted associations of plasma vitamin C with prostate cancer in the dataset from the Prostate Cancer Association Group to Investigate Cancer Associated Alterations in the Genome (PRACTICAL).

## Data Availability

All the data used in the present study had been publicly available, and the source of the data had been described in the main text.

## Abbreviations

MR: Mendelian randomization
GWAS: Genome-wide association study
OR: Odds ratio
RCT: Randomized controlled trials
SNPs: Single-nucleotide polymorphisms
EPIC: European Prospective Investigation into Cancer and Nutrition
ILCCO: International Lung Cancer Consortium
PRACTICAL: Prostate Cancer Association Group to Investigate Cancer Associated Alterations in the Genome
BCAC: Breast Cancer Association Consortium
SD: Standard deviation
CI: Confidence intervals
IVW: Inverse-variance-weighted
MR-PRESSO: MR pleiotropy residual sum and outlier
MR-RAPS: MR Robust Adjusted Profile Score
MBE: Mode-based estimation
