# Supplementary material for: Circulating vitamin C concentration and risk of cancers: a Mendelian randomization study
Source: BMC Med. 2021 Jul 30;19:171. doi: 10.1186/s12916-021-02041-1 (PMC8323227; doi:10.1186/s12916-021-02041-1)
Supplement: Supplementary file 1 — Additional file 1: Supplemental Table 1. The plasma vitamin C-related genetic variants used for the MR analyses. Supplemental Table 3. Characteristics of the genetic variants that were used as the instrumental variables for plasma vitamin C concentration. Supplemental Table 4. Genetic correlation between vitamin C and site-specific cancers, estimated through linkage disequilibrium score regression. Supplemental Table 5. Mendelian randomization estimates of the association between genetically predicted plasma vitamin C concentration and risk of secondary cancer outcomes based on cancer subtypes. Supplemental Table 6. Multivariate MR analysis exploring causal association between circulating Vitamin C and lung cancer with adjustment for smoking. [file 12916_2021_2041_MOESM1_ESM.docx]

| **Supplemental Table 1. The plasma vitamin C-related genetic variants used for the MR analyses** | | | | | | | |
| --- | --- | --- | --- | --- | --- | --- | --- |
| **Study** | Target SNP | Proxy SNP | Target A1 | Target A2 | Proxy SNP A1 | Proxy SNP A2 | Included for analysis |
| **UK Biobank** | rs6693447 | NULL | G | T | NULL | NULL | YES |
|  | rs13028225 | NULL | C | T | NULL | NULL | YES |
|  | rs33972313 | NULL | T | C | NULL | NULL | YES |
|  | rs10051765 | NULL | C | T | NULL | NULL | YES |
|  | rs7740812 | NULL | A | G | NULL | NULL | YES |
|  | rs117885456 | NULL | A | G | NULL | NULL | YES |
|  | rs2559850 | NULL | G | A | NULL | NULL | YES |
|  | rs10136000 | NULL | A | G | NULL | NULL | YES |
|  | rs56738967 | NULL | C | G | NULL | NULL | YES |
|  | rs9895661 | NULL | C | T | NULL | NULL | YES |
| **BCAC** | rs6693447 | NULL | G | T | NULL | NULL | YES |
|  | rs13028225 | NULL | C | T | NULL | NULL | YES |
|  | rs33972313 | NULL | T | C | NULL | NULL | YES |
|  | rs10051765 | NULL | C | T | NULL | NULL | YES |
|  | rs7740812 | NULL | A | G | NULL | NULL | YES |
|  | rs10136000 | NULL | A | G | NULL | NULL | YES |
|  | rs56738967 | NULL | C | G | NULL | NULL | YES |
|  | rs9895661 | NULL | T | C | NULL | NULL | YES |
|  | rs117885456 | NULL | A | G | NULL | NULL | YES |
|  | rs2559850 | NULL | A | G | NULL | NULL | YES |
| **PRACTICAL** | rs6693447 | NULL | T | G | NULL | NULL | YES |
|  | rs13028225 | NULL | T | C | NULL | NULL | YES |
|  | rs33972313 | NULL | T | C | NULL | NULL | YES |
|  | rs10051765 | NULL | T | C | NULL | NULL | YES |
|  | rs7740812 | NULL | A | G | NULL | NULL | YES |
|  | rs117885456 | NULL | A | G | NULL | NULL | YES |
|  | rs2559850 | NULL | A | G | NULL | NULL | YES |
|  | rs10136000 | NULL | A | G | NULL | NULL | YES |
|  | rs56738967 | NULL | C | G | NULL | NULL | YES |
|  | rs9895661 | NULL | T | C | NULL | NULL | YES |
| **OCAC** | rs10051765 | NULL | C | T | NULL | NULL | YES |
|  | rs10136000 | NULL | A | G | NULL | NULL | YES |
|  | rs13028225 | NULL | T | C | NULL | NULL | YES |
|  | rs33972313 | NULL | C | T | NULL | NULL | YES |
|  | rs56738967 | NULL | C | G | NULL | NULL | YES |
|  | rs6693447 | NULL | T | G | NULL | NULL | YES |
|  | rs7740812 | rs9689405 | A | G | C | T | YES |
|  | rs9895661 | NULL | T | C | NULL | NULL | YES |
| **Supplemental Table 1. The plasma vitamin C-related genetic variants used for the MR analyses (continued)** | | | | | | | |
| **Study** | Target SNP | Proxy SNP | Target A1 | Target A2 | Proxy SNP A1 | Proxy SNP A2 | Included for analysis |
|  | rs117885456 | NR | A | G | NR | NR | NO |
|  | rs2559850 | NR | A | G | NR | NR | NO |
| **ILCCO** | rs10051765 | NULL | C | T | NULL | NULL | YES |
|  | rs10136000 | NULL | A | G | NULL | NULL | YES |
|  | rs117885456 | NULL | A | G | NULL | NULL | YES |
|  | rs13028225 | NULL | T | C | NULL | NULL | YES |
|  | rs2559850 | NULL | A | G | NULL | NULL | YES |
|  | rs33972313 | NULL | C | T | NULL | NULL | YES |
|  | rs56738967 | NULL | C | G | NULL | NULL | YES |
|  | rs6693447 | NULL | T | G | NULL | NULL | YES |
|  | rs7740812 | NULL | G | A | NULL | NULL | YES |
|  | rs9895661 | NULL | T | C | NULL | NULL | YES |

NR: not reported in the original data source

**Supplemental Table 3. Characteristics of the genetic variants that were used as the instrumental variables for plasma vitamin C concentration***

| SNP | Beta | SE | EAF | Effect allele | Other  allele | | p-value | Sample size | R^2^ |
| --- | --- | --- | --- | --- | --- | --- | --- | --- | --- |
| rs6693447 | 0.039 | 0.006 | 0.551 | T | G | 6.25×10^-10^ | | 52,018 | 8.12E-04 |
| rs13028225 | 0.102 | 0.009 | 0.857 | T | C | 2.38×10^-30^ | | 52,018 | 2.46E-03 |
| rs33972313 | 0.36 | 0.018 | 0.968 | C | T | 4.61×10^-90^ | | 52,018 | 7.63E-03 |
| rs10051765 | 0.039 | 0.007 | 0.342 | C | T | 3.64×10^-9^ | | 52,018 | 5.96E-04 |
| rs7740812 | 0.038 | 0.006 | 0.594 | G | A | 1.88×10^-9^ | | 52,018 | 7.71E-04 |
| rs117885456 | 0.078 | 0.012 | 0.087 | A | G | 1.70×10^-11^ | | 52,018 | 8.12E-04 |
| rs2559850 | 0.058 | 0.006 | 0.598 | A | G | 6.30×10^-20^ | | 52,018 | 1.79E-03 |
| rs10136000 | 0.04 | 0.007 | 0.283 | A | G | 1.33×10^-8^ | | 52,018 | 6.27E-04 |
| rs56738967 | 0.041 | 0.007 | 0.321 | C | G | 7.62×10^-10^ | | 52,018 | 6.59E-04 |
| rs9895661 | 0.063 | 0.008 | 0.817 | T | C | 1.05×10^-14^ | | 52,018 | 1.19E-03 |

SNP, single nucleotide polymorphism; SE, standard error; EAF, effect allele frequency; Beta coefficients are in standard deviation (SD) unit per allele; effect allele is the vitamin C-raising allele.

*Source: Zheng, et al., 2020.^1^

**Supplemental Table 4. Genetic correlation between vitamin C and site-specific cancers, estimated through linkage disequilibrium score regression.**

| Traits | Genetic correlation | SE | p-value |
| --- | --- | --- | --- |
| Bronchus and lung cancer | -0.4346 | 0.1687 | 0.010 |
| Breast cancer | -0.0972 | 0.0802 | 0.226 |
| Prostate cancer | -0.0985 | 0.0870 | 0.258 |
| Colon cancer | -0.1627 | 0.1262 | 0.197 |
| Rectal cancer | -0.0767 | 0.1631 | 0.638 |
| Smoking initiation | -0.2402 | 0.04306 | <0.0001 |

**Supplemental Table 5. Mendelian randomization estimates of the association between genetically predicted plasma vitamin C concentration and risk of secondary cancer outcomes based on cancer subtypes**

| **Cancer type** | IVW | MR-Egger | Weighted median-based | MR-PRESSO | MR-MBE | MR-Robust | MR-RAPS | MR-PRESSO Test | MR-Egger regression | Cochran’s Q test |  |
| --- | --- | --- | --- | --- | --- | --- | --- | --- | --- | --- | --- |
|  | OR (95% CI) | OR (95% CI) | OR (95% CI) | OR (95% CI) | OR (95% CI) | OR (95% CI) | OR (95% CI) | p-value | p-value | q-value |  |
| **BCAC** |  |  |  |  |  |  |  |  |  |  |  |
| Breast cancer |  |  |  |  |  |  |  |  |  |  |  |
| ER-positive | 1.07(0.94,1.21) | 1.03(0.82,1.23) | 1.07(0.96,1.18) | 1.07(0.94,1.21) | 1.05(0.94,1.16) | 1.06(0.98,1.15) | 1.05(0.95,1.17) | 0.032 | 0.478 | 0.012 |  |
| ER-negative | 1.01(0.89,1.15) | 1.05(0.84,1.29) | 1.04(0.88,1.21) | 1.01(0.89,1.15) | 1.05(0.88,1.24) | 1.02(0.91,1.13) | 1.02(0.89,1.16) | 0.437 | 0.712 | 0.418 |  |
| **ILCCO** |  |  |  |  |  |  |  |  |  |  |  |
| Lung cancer |  |  |  |  |  |  |  |  |  |  |  |
| Adenocarcinoma | 1.27(0.85,1.89) | 1.18(0.60,2.34) | 1.05(0.71,1.54) | 1.27(0.85,1.89) | 1.08(0.72,1.61) | 1.19(0.85,1.69) | 1.17(0.83,1.66) | 0.077 | 0.810 | 0.060 |  |
| Squamous cell carcinoma | 0.96(0.67,1.37) | 1.14(0.63,2.05) | 0.98(0.66,1.44) | 0.96(0.67,1.37) | 0.95(0.63,1.41) | 0.94(0.73,1.20) | 0.94(0.68,1.32) | 0.254 | 0.485 | 0.180 |  |
| BCAC, the Breast Cancer Association Consortium; ILCCO, International Lung Cancer Consortium;  IVW, Inverse Variance Weighted; PRESSO, Pleiotropy Residual Sum and Outlier; MBE, Mode Based Estimation; RAPS, Robust Adjusted Profile Score. | | | | | | | | | | | |

**Supplemental Table 6. Multivariate MR analysis exploring causal association between circulating Vitamin C and lung cancer with adjustment for smoking.**

| **Vitamin C to lung cancer** | | | | | |
| --- | --- | --- | --- | --- | --- |
|  | OR | se | 95% CI | | p |
| multivariate MR-IVW | 1.10 | 0.07 | 0.95 | 1.26 | 0.214 |
| multivariate MR-Egger | 1.09 | 0.14 | 0.83 | 1.43 | 0.546 |
| **Lung cancer to vitamin C** | | | | | |
|  | Beta | se | 95% CI | | p |
| multivariate MR-IVW | -0.015 | 0.02 | -0.034 | 0.004 | 0.111 |
| multivariate MR-Egger | -0.015 | 0.02 | -0.034 | 0.004 | 0.112 |

IVW, Inverse Variance Weighted.
