# Supplementary material for: Circulating vitamin C concentration and risk of cancers: a Mendelian randomization study
Source: BMC Med. 2021 Jul 30;19:171. doi: 10.1186/s12916-021-02041-1 (PMC8323227; doi:10.1186/s12916-021-02041-1)
Supplement: Supplementary file 3 — Additional file 3: Table S1-S2 & Figure S1-S8. Supplemental methods and results for the systematic review and meta-analysis. TableS1. PubMed search strategy. TableS2. Characteristics of the included prospective studies. FigS1. Flow diagrams of the literature research. FigS2. The association of dietary and supplemental vitamin C intakes with incident lung cancer. FigS3. The association of dietary, supplemental and total vitamin C intakes with incident breast cancer. FigS4. The association of dietary, supplemental and total vitamin C intakes with incident prostate cancer. FigS5. The association of dietary vitamin C intakes with incident colorectal cancer. FigS6. The association of supplemental vitamin C intakes with incident colorectal cancer. FigS7. The association of total vitamin C intakes with incident colorectal cancer. FigS8. Funnel plot for associations of vitamin C intakes with cancers, with Egger’s test adopted to examine the publication bias. [file 12916_2021_2041_MOESM3_ESM.docx]

**Additional file 3. Supplemental methods and results for the systematic review and meta-analysis**

**Search strategy and inclusion criteria**

A systematic literature search was performed in PubMed, from its inception to Feb 18, 2021. The search strategies were listed in the Table S1, without any restriction on language. After removing duplicates, two of the authors (YF and XL) screened the articles independently, and discrepancies were resolved by group discussion with a third investigator (FX). Cohort studies were included if they met the following criteria: (1) the study was prospective cohort study (cohort, case-cohort, or nested case-control study); (2) the exposure was vitamin C intake (dietary, supplemental or total vitamin C intakes); (3) the outcome was the risk of 4 site-specific cancers of interest, namely lung, breast, prostate and colorectal cancer. Studies were excluded if (1) the study has a cross-sectional design; (2) the study did not report the risk estimates (RR, HR or OR) comparing the highest category of vitamin C exposure versus the lowest category, with the corresponding 95% CIs. When more than one articles were available on the same association base on the same cohort, we included the one with the largest number of cases. The study selection process of prospective cohort studies was shown in Figure S1.

**Data extraction**

Data extraction was done independently by two investigators (XL and ZM). From each eligible article, we recorded the first author, year of publication, study design, exposures, outcomes, number of cases, number of controls or cohort size, and multivariate adjusted risk estimates together with the corresponding 95% CIs. The basic characteristics of the included studies are listed in the Table S2.

**Data synthesis and analysis**

We used Stata 15.0 (StataCorp) for all analyses: a 2-tailed p value <0.05 was regarded as statistically significant. The RR was used as the common risk estimate, the HR and OR was treated as the RR directly. If a study reported stratified risk estimates (e.g., women vs. men, , premenopausal vs. postmenopausal), we pooled the risk estimates by a fixed-effects model to obtain a cohort-specific risk estimate, assuming an internal consistency within a single study.

We performed random-effects meta-analyses, assuming that biological effects of vitamin C intakes among different populations would vary randomly, to pool RRs and 95% Cis for the highest versus lowest categories of exposure. Heterogeneity between studies was evaluated by I2 statistics. I2 values of 25 %, 50%, and 75% corresponded to cutoff points for low, moderate, and high degrees of heterogeneity, respectively. We examined publication bias of included cohort studies using Begg’s funnel plot and Egger’s regression test, with p<0.05 to indicate significant asymmetry. The results were shown in Supplemental Figure S2-S8.

**Content**

**Additional file 3-Table S1.** PubMed search strategy for prospective studies of association between vitamin C intakes (dietary, supplemental or total) and the primary cancer outcomes (lung, breast, prostate and colorectal cancer)*

**Additional file 3-Table S2.** Characteristics of the included prospective studies of the association between vitamin C intakes (dietary, supplemental or total) and the cancer outcomes (lung, breast, prostate and colorectal cancer)

**Additional file 3-Figure S1.** Flow diagrams of the literature research

**Additional file 3-Figure S2.** The association of dietary and supplemental vitamin C intakes with incident lung cancer

**Additional file 3-Figure S3.** The association of dietary, supplemental and total vitamin C intakes with incident breast cancer

**Additional file 3-Figure S4.** The association of dietary, supplemental and total vitamin C intakes with incident prostate cancer

**Additional file 3-Figure S5.** The association of dietary vitamin C intakes with incident colorectal cancer

**Additional file 3-Figure S6.** The association of supplemental vitamin C intakes with incident colorectal cancer

**Additional file 3-Figure S7.** The association of total vitamin C intakes with incident colorectal cancer

**Additional file 3-Figure S8.** Funnel plot for associations of vitamin C intakes with lung cancer (a), breast cancer (b), prostate cancer (c), and colorectal cancer (d). with Egger’s test adopted to examine the publication bias.

**Additional file 3-Table S1. PubMed search strategy for prospective observational studies of association between vitamin C intakes (dietary, supplemental or total) and the primary cancer outcomes (lung, breast, prostate and colorectal cancer)***

| Search strategy | Search terms | No. of studies identified | Complementary search terms | No. of studies identified | No. of studies identified from the bibliographies of relevant articles. | No. of studies included |
| --- | --- | --- | --- | --- | --- | --- |
| Strategy 1 | ((vitamin C[Title/Abstract]) OR (ascorbic acid[Title/Abstract])) AND ((lung[Title/Abstract]) OR (bronchus[Title/Abstract])) AND ((cancer[Title/Abstract]) OR (neoplasm[Title/Abstract]) OR (adenocarcinoma[Title/Abstract]) OR (carcinoma[Title/Abstract]) OR (tumor[Title/Abstract]) OR (tumour[Title/Abstract])) | 382 | (((Ascorbic Acid[MeSH Terms]) ) AND (Lung Neoplasms[MeSH Terms])) AND (humans[Filter])) (("1900/1/1"[Date - Publication] : "2021/1/18"[Date - Publication])) | 143 | 0 | 10 |
| Strategy 2 | ((vitamin C[Title/Abstract]) OR (ascorbic acid[Title/Abstract])) AND ((breast[Title/Abstract]) OR (mammary[Title/Abstract]))) AND ((cancer[Title/Abstract]) OR (neoplasm[Title/Abstract]) OR (adenocarcinoma[Title/Abstract]) OR (carcinoma[Title/Abstract]) OR (tumor[Title/Abstract]) OR (tumour[Title/Abstract])) | 469 | ((Ascorbic acid) AND (Breast Neoplasms) AND (humans[Filter])) AND (("1900/1/1"[Date - Publication] : "2021/2/18"[Date - Publication])) | 260 | 3 | 14 |
| Strategy 3 | ((vitamin C[Title/Abstract]) OR (ascorbic acid[Title/Abstract])) AND (prostate[Title/Abstract]) AND ((cancer[Title/Abstract]) OR (neoplasm[Title/Abstract]) OR (adenocarcinoma[Title/Abstract]) OR (carcinoma[Title/Abstract]) OR (tumor[Title/Abstract]) OR (tumour[Title/Abstract])) | 194 | ((Ascorbic Acid[MeSH Terms]) AND (Prostatic Neoplasms[MeSH Terms]) AND (humans[Filter])) AND (("1900/1/1"[Date - Publication] : "2021/1/18"[Date - Publication])) | 86 | 1 | 6 |
| Strategy 4 | ((vitamin C[Title/Abstract]) OR (ascorbic acid[Title/Abstract])) AND ((colorectum[Title/Abstract]) OR (Colorectal[Title/Abstract]) OR (colon [Title/Abstract]) OR (colonic [Title/Abstract]) OR (rectal[Title/Abstract]) OR (tectum[Title/Abstract])) AND ((cancer[Title/Abstract]) OR (neoplasm[Title/Abstract]) OR (adenocarcinoma[Title/Abstract]) OR (carcinoma[Title/Abstract]) OR (tumor[Title/Abstract]) OR (tumour[Title/Abstract])) | 413 | ((Ascorbic Acid[MeSH Terms]) AND (Colorectal Neoplasms[MeSH Terms])) AND (("1900/1/1"[Date - Publication] : "2021/2/18"[Date - Publication])) AND (humans[Filter]) | 159 | 2 | 14 |

*We systematically searched PubMed from inception to Feb 18, 2021. The search was restricted to human studies, without restriction on language.

**Additional file 3-Table S2. Characteristics of the included prospective studies of the association between vitamin C intakes (dietary, supplemental or total) and the cancer outcomes (lung, breast, prostate and colorectal cancer)**

| **First author, year of publication, cohort, county** | **Study design** | **Baseline years** | **Follow-up, y** | **Age (range or mean), y** | **Women**  **(%)** | **Exposures**  **(dietary, supplemental or total vitamin C intake)** | **Outcomes and No. of cases** | **No. of controls/ cohort size** | **Multivariate adjusted risk estimates (RR/HR/OR) and 95% CIs** | **Adjustment model** |
| --- | --- | --- | --- | --- | --- | --- | --- | --- | --- | --- |
| Narita et al. 2018, JPHC, Japan ^49^ | Prospective cohort | 1990-1994 | 18 | 40-69 | 52.1 | Diet | Lung cancer (n=1690) | 79705 | Men: 1.02 (0.79, 1.32)  Women: 1.37 (0.92, 2.05) | Age, study area, smoking status, alcohol consumption, vitamin supplements use and energy-adjusted intakes of fish, isoflavone, vegetables and fruits. |
| Voorrips et al. 2000, NCSDC, Netherlands ^50^ | Prospective cohort | 1986 | 6.3 | 55-69 | 0 | Diet | Lung cancer  (n=939) | 58279 | 0.77 (0.54, 1.08) | Age, family history, smoking, SES, folate, energy |
| Shibata et al. 1992, LWS, USA ^51^ | Prospective cohort | 1981 | 8 | Women: 73.8  Men: 74.9 | NR | Diet/Supplement | Lung cancer (n=164)  Breast cancer (n=219)  Prostate cancer  (n=208)  Colon cancer (n=202) | 11580 | **Dietary vitamin C and lung cancer**  Women: 0.56 (0.31, 1.02)  Men: 1.11 (0.68, 1.81)  **Supplemental vitamin C and lung cancer**  Women: 0.72 (0.45, 1.15)  Men: 1.03 (0.68, 1.55)  **Dietary vitamin C and Breast cancer**  Women: 0.86 (0.63-1.18)  **Supplemental vitamin C and lung cancer**  Women: 0.93 (0.71, 1.23)  **Dietary vitamin C and prostate cancer**  0.96 (0.68-1.35)  **Supplemental vitamin C and prostate cancer**  1.00 (0.76, 1.32)  **Dietary vitamin C and lung cancer**  Women: 0.56 (0.31, 1.02)  Men: 1.11 (0.68, 1.81)  **Supplemental vitamin C and lung cancer**  Women: 0.72 (0.45, 1.15)  Men: 1.03 (0.68, 1.55)  **Dietary vitamin C and colon cancer**  Women: 0.61 (0.38, 0.99)  Men: 1.15 (0.70, 1.88)  **Supplemental vitamin C and colon cancer**  Women: 0.67 (0.45, 0.99)  Men: 0.92 (0.62, 1.38) | Age and smoking. |
| Slatore et al. 2007, VITAL, USA ^52^ | Prospective cohort | 2002 | 4.05 | 50-76 | 52.0% | Supplement | Lung cancer  (n=521) | 77126 | 0.97 (0.76, 1.23) | smoking, age, and sex |
| Roswall et al. 2010, Denmark ^53^ | Prospective cohort | 1993-1997 | 10.6 | 50-64 | 52.3% | Diet/Supplement | Lung cancer (n=721) | 55557 | Diet: 0.76 (0.58, 0.99)  Supplement: 1.23 (0.93, 1.62) | intake of folate, vitamin E, and Beta-carotene, smoking status, smoking duration, smoking intensity, possible cessation and when, passive smoking, work exposure. |
| Yuan et al. 2003, SCHS, Singapore ^54^ | Prospective cohort | 1993-1998 | 8 | 45-74 | NR | Diet | Lung cancer (n=482) | 62392 | 0.81 (0.59, 1.09) | age, sex, dialect group, year of interview, level of education, BMI, r number of cigarettes smoked per day, number of years of smoking, and number of years since quitting smoking for former smokers |
| Yong et al. 1997, NHANES, USA ^55^ | Prospective  cohort | 1971-1975 | 19 | 25-74 | 60.6 | Diet | Lung cancer  (n=248) | 10068 | 0.66 (0.45, 0.96) | Sex, race, educational attainment, nonrecreational activity level, BMI, family history, smoking status, total calorie intake, and alcohol intake. |
| Takata et al. 2013, SMHS, China ^56^ | Prospective cohort | 2002-2006 | 5.5 | 40-74 | NR | Diet | Lung cancer (n=359) | 61491 | 0.84 (0.61, 1.16) | Age, years of smoking, the number of cigarettes smoked per day, current smoking status, total caloric intake, education, BMI category, ever consumption of tea, history of chronic bronchitis, and family history of lung cancer among first-degree relatives. |
| Nagel et al. 2010, EPIC, EU ^57^ | Prospective  cohort | 1992 | 8.8 | 35-70 | 100 | Diet | Breast cancer (n=6478) | 288776 | Premenopausal: 1.12 (0.92-1.36)  Postmenopausal: 0.98 (0.87,1.11) | Energy from protein and carbohydrates, saturated fatty acid, monounsaturated fatty acids, polyunsaturated fatty acids, alcohol intake, weight, height, age at menarche, parity, age at first full-term pregnancy, use of hormone therapy at recruitment, smoking status, physical activity index, education. |
| Zhang et al. 1999, NHS, USA ^15^ | Prospective  cohort | 1980 | 14 | 33-60 | 100 | Diet/Total | Breast cancer (n=2697) | 83234 | **Dietary vitamin C intake:**  Premenopausal: 1.01 (0.81, 1.26)  Postmenopausal: 1.06 (0.91, 1.22)  **Total vitamin C intake:**  Premenopausal: 1.01 (0.81, 1.26)  Postmenopausal: 0.99 (0.85, 1.14) | Age, length of follow-up, total energy intake, parity, age at first birth, age at menarche, history of breast cancer in mother or a sister, history of benign breast disease, alcohol intake, body mass index, and height. |
| Verhoeven et al. 1997, NCS, Netherlands ^58^ | Case cohort | 1986 | 4.3 | 55-69 | 100 | Diet | Breast cancer  (n=650) | 1812 | 0.77 (0.55-1.08) | age, energy intake, alcohol intake, history of benign breast disease, maternal breast cancer, breast cancer in sister(s), age at menarche, age at menopause, age at first birth, parity. |
| Kushi et al. 1996, IWHS, USA ^12^ | Prospective  cohort | 1986 | 7 | 55-69 | 100 | Diet/Supplement/Total | Breast cancer (n=879) | 34387 | Diet: 1.06 (0.77, 1.47)  Supplement: 0.77 (0.50, 1.17)  Total: 0.88 (0.70-1.11) | Age, age at menarche, age at menopause, age at first live birth, parity, BMI at time of baseline questionnaire, BMI at age 18, family history of breast cancer, history of benign breast disease, alcohol intake, and education. |
| Hunter et al. 1993, USA ^59^ | Prospective  cohort | 1980 | 8 | 30-59 | 100 | Total | Breast cancer  (n=1439) | 89494 | 1.03 (0.87-1.21) | Age, length of follow-up, energy intake, parity, age at first birth, age at menarche, history of breast cancer in mother or a sister, menopausal status, BMI, alcohol intake, history of benign breast disease. |
| Michels et al. 2001, SMSC, Sweden ^60^ | Prospective  cohort | 1987-1990 | 10 | 40-76 | 100 | Diet | Breast cancer (n=1271) | 59036 | 0.94 (0.78-1.14) | Age, family history of breast cancer, height, BMI, education, parity, age at ﬁrst birth, total caloric intake and intake of alcohol, ﬁber and monounsaturated fatty acids. |
| Cui et al. 2008, WHI, USA ^61^ | Prospective  cohort | 1993-1998 | 7.6 | 50-79 | 100 | Diet/Supplement/Total | Breast cancer  (n=2879) | 84805 | Diet: 1.06 (0.92, 1.22)  Supplement: 1.16 (1.04, 1.30)  Total:1.18 (1.04, 1.34) | Energy intake, age at baseline, ethnicity, educational level, age at menarche, age at menopause, parity, age at first full-term pregnancy, oral contraceptive use, postmenopausal hormone use, BMI, physical activity, alcohol drinking, dietary folate intake, tobacco smoking, hysterectomy, bilateral oophorectomy, history of benign breast disease, and family history of breast cancer. |
| Rohan et al. 1993, NBSS, Canada ^14^ | Nested case control | 1982 | 5 | 40-59 | 100 | Diet/supplement | Breast cancer (n=519) | 1182 | Diet:0.88 (0.62, 1.26)  Supplement:1.46 (1.05, 2.01) | Age, energy intake, age at menarche, surgical menopause, age at first livebirth, years of education, family history of breast cancer, and history of benign breast disease. |
| Cadeau et al. 2016, E3N, France ^17^ | Prospective  cohort | 1990 | 10.1 | 40-65 | 100 | Diet/Supplement | Breast cancer  (n=2482) | 57403 | Diet:0.91 (0.81, 1.03)  Supplement: 1.08 (0.94, 1.23) | Age, year of birth, BMI, use of oral contraceptives before menopause, use of menopausal hormone therapy, parity and age at first full-term pregnancy, age at menarche, age at menopause, total energy intake without alcohol, current vitamin D supplementation, alcohol consumption, physical activity, personal history of benign breast disease, smoking status, mammography in the previous follow-up period, family history of breast cancer in first-degree relatives, and educational level, use of micronutrient supplements other than vitamin C, calcium, or vitamin D. |
| Pantovas et al. 2015,Rotterdam Study, Netherlands ^62^ | Prospective  cohort | 1989-1993 | 17 | >55 | 100 | Diet | Breast cancer  (n=199) | 3209 | 0.88 (0.63-1.25) | Age, BMI, educational level, family history of breast cancer, smoking status and alcohol consumption, use of multivitamin supplement. |
| Roswall et al. 2010, Denmark ^63^ | Prospective  cohort | 1993-1997 | 10.6 | 50-64 | 100 | Diet/Supplement/Total | Breast cancer  (n=1072) | 26224 | Diet: 1.15 (0.92, 1.44)  Supplement: 0.96 (0.77, 1.21)  Total: 1.11 (0.88-1.40) | Total intake of the three other micronutrients as well as dietary intake for the supplemental intake and supplemental intake for the dietary intake and further for alcohol intake, BMI, hormone replacement therapy (HRT) use, duration of HRT use, number of births, parity/nulliparity, age at first birth and school education. |
| Cho et al. 2003, NHSII, USA ^64^ | Prospective cohort | 1991 | 8 | 25-42 | 100 | Diet/Total | Breast cancer (n=714) | 90655 | Diet:1.30 (1.00-1.69)  Total: 0.96 (0.75-1.21) | Age in months at start of follow-up and calendar year of the current questionnaire cycle and was simultaneously adjusted for smoking, height, parity and age at first birth, BMI, age at menarche, family history of breast cancer, history of benign breast disease, oral contraceptive use, menopausal status, alcohol intake, energy, and animal fat. |
| Kirsh et al. 2006, PLCO, USA ^65^ | Prospective cohort | 1993-2001 | 8 | 55-74 | 0 | Diet/Supplement | Prostate cancer  (n=1338) | 29361 | Diet: 1.00 (0.83, 1.22)  Supplement: 1.01 (0.87, 1.17) | Age, total energy, race, study center, family history of prostate cancer, body mass index, smoking status, physical activity, total fat intake, red meat intake, history of diabetes, aspirin use, and number of screening examinations during the follow-up period |
| Roswall et al. 2010, DPCS, Denmark ^66^ | Prospective cohort | 1993 | 14.3 | 50-64 | 0 | Diet/Supplement/total | Prostate cancer (n=1571) | 26856 | Diet: 0.92 (0.77-1.09)  Supplement: 0.94 (0.76, 1.16)  Total: 0.95 (0.78-1.15) | Intake of the three other micronutrients as well as dietary intake for the supplemental intake and supplemental intake for the dietary intake and further for height, weight, education, intake of red meat, alcohol consumption, selenium intake |
| Daviglus et al. 1996, WES, USA ^67^ | Prospective cohort | 1957-1958 | 30 | 40-55 | 0 | Diet | Prostate cancer (n=132) | 1899 | 1.27 (0.75-2.14) | Age, number of cigarettes smoked per day, dietary cholesterol and saturated fat, ethanol intake, total energy intake, and occupation. |
| Schuurman et al. 2002, NCS, Netherlands ^68^ | Prospective cohort | 1986 | 6.3 | 55-69 | 0 | Diet | Prostate cancer (n=642) | 58279 | 1.15 (0.87-1.52) | age, family history of prostate cancer, socioeconomic status, and alcohol from white or fortified wine |
| Ruder et al. 2011, NIH-AARP, USA ^69^ | Prospective cohort | 1995-1996 | NR | 62.8 | 41.5 | Diet | Colon cancer (n=2794)  Rectal cancer  (n=979) | 292797 | Dietary vitamin C intake and colon cancer:  0.96 (0.85, 1.08)  Dietary vitamin C intake and rectal cancer:  0.96 (0.77, 1.19) | Energy at ages 12–13 y, energy in recent adulthood, nutrient of interest in recent adulthood, age at completion of risk-factor questionnaire, sex, BMI, race, education, physical activity, alcohol consumption, smoking, use of nonsteroidal antiinflammatory drugs, use of hormone replacement therapy, and self-report of a first-degree relative with a history of colon cancer. |
| Egnell et al. 2017,  NutriNet-Sante, France ^70^ | Prospective cohort | 2009 | 7 | 57.1 | NR | Diet/Supplement/Total | Colorectal cancer  (n=120) | 38812 | Diet: 0.66 (0.38, 1.16)  Supplement: 0.45 (0.14, 1.41)  Total:0.60 (0.33, 1.09) | age, sex, BMI, height, physical activity, smoking status, number of cigarette packs per year, numbers of dietary records, alcohol intake, energy intake, family history of cancer and educational level. |
| Wu et al. 1987, USA ^71^ | Prospective cohort | 1981 | 4.5 | NR | NR | Diet/Supplement | Colorectal cancer (n=126) | 11888 | **Diet:**  Women 0.5 (0.3, 0.9)  Men 0.88 (0.5, 1.7)  **Supplement:**  Women 0.82 (0.4, 1.6)  Men 1.11 (0.6, 2.2) | Age |
| Sellers et al. 1998, IWHS, USA ^72^ | Prospective cohort | 1986 | 9 | 55-69 | 100 | Diet/Supplement/Total | Colon cancer (n=241) | 35216 | **Diet:**  No family history 0.8 (0.6, 1.2)  Positive family history 1.1 (0.5, 2.2)  **Supplement:**  No family history 0.7 (0.5, 1.4)  Positive family history 0.7 (0.4, 1.3  **Total:**  No family history 0.7 (0.5, 1.0)  Positive family history 0.8 (0.4, 1.4) | Age at baseline, total energy intake, and history of rectal colon polyps |
| Zheng et al. 1998, IWHS, USA ^73^ | Prospective cohort | 1986 | 9 | 55-69 | 100 | Diet | Rectal cancer  (n=144) | 34702 | 0.84 (0.56, 1.26) | Age, smoking status. pack-years of smoking, use of hormone replacement therapy, and total energy intake. |
| Malila et al. 2002, ATBC, Finland ^74^ | Prospective  cohort | 1994 | 8 | 50-69 | 0 | Diet | Colorectal cancer (n=184) | 29561 | 1.16 (0.77-1.76) | Age, body mass index, alcohol intake, serum cholesterol, physical activity, cigarettes, trial supplementation. |
| Roswall et al. 2010, Denmark ^75^ | Prospective  cohort | 1993-1997 | 10.6 | 50-64 | 52.3 | Diet/Supplement/Total | Colon cancer (n=465)  Rectal cancer (n=283) | 56332 | **Diet:**  Colon cancer 1.15 (0.83, 1.60)  Rectal cancer 1.33 (0.87, 2.03)  **Supplement:**  Colon cancer 1.05 (0.73, 1.49)  Rectal cancer 1.51 (0.98, 2.33)  **Total:**  Colon cancer 1.00 (0.72, 1.39)  Rectal cancer 1.40 (0.92, 2.14) | Intake of the three other micronutrients as well as dietary intake for the supplemental intake and supplemental intake for the dietary intake and further for body mass index, education, alcohol, red meat intake, processed meat intake, smoking status, leisure time physical activity, calcium consumption, HRT use. |
| Vece et al. 2015, I-EPIC, Italy^76^ | Prospective cohort | 1993-1998 | 10-13 | Mean: 49.8-51.4 | 68.6 | Diet | Colorectal cancer （n=434）  Colon cancer (n=325)  Rectal cancer (n=109) | 45194 | Colorectal cancer 0.83 (0.61-1.11)  Colon cancer 0.75 (0.54-1.06)  Rectal cancer 1.10 (0.60-2.01) | Age, sex, BMI, height, smoking status, education, intakes of alcohol, total physical activity, fiber, non alcohol energy intake, red meat, processed meat, calcium and dietary fiber |
| Shin et al. 2006, SWHS, China ^77^ | Prospective cohort | 1997-2000 | 5.74 | 40-70 | 100 | Diet | Colorectal cancer  (n=283)  Conlon cancer (n=129)  Rectal cancer (n=91) | 73314 | Colon cancer:   1. (0.5, 1.9)   Rectal cancer:  1.2 (0.6, 2.4)  Colorectal cancer:  1.1 (0.7, 1.6) | Age, menopausal status, education, cigarette smoking, alcohol consumption, exercise, family history of colorectal cancer among 1st degree relatives, vitamin supplements use and calorie intake. |
| Leenders et al. 2014, EPIC, EU ^78^ | Nested case-control | 1992 | 1.0-8.5 | 50-67 | 52.1 | Diet | Colon cancer (n=898)  Rectal cancer (n=501) | 1399 | Colon cancer  0.76 (0.57, 1.01)  Rectal cancer  1.01 (0.68, 1.51) | matching factors and physical activity, smoking status, number of cigarettes smoked per day, smoking duration, time  since stopped smoking, alcohol consumption at baseline, waist circumference and highest level of education  matching factors and physical activity, smoking status, number of cigarettes smoked per day, smoking duration, time  since stopped smoking, alcohol consumption at baseline, waist circumference and highest level of education  matching factors and physical activity, smoking status, number of cigarettes smoked per day, smoking duration, time  since stopped smoking, alcohol consumption at baseline, waist circumference and highest level of education  Matching factors and physical activity, smoking status, number of cigarettes smoked per day, smoking duration, time since stopped smoking, alcohol consumption at baseline, waist circumference and highest level of education |
|  |  |  |  |  |  |  |  |  |  |  |
| Lin et al. 2009, WACS, USA ^13^ | RCT | 1996-1997 | 9.4 | 60.4 | 100 | Supplements | Lung cancer (n=74);  Breast cancer (n=257);  Colorectal cancer (n=44) | 7627 | Lung cancer 1.84 (1.14, 2.97);  Breast cancer 1.11 (0.87, 1.41);  Colorectal cancer 0.76 (0.42, 1.38) | None |
| Gaziano, et al. 2009, PHSII, USA ^21^ | RCT | 1997 | 8.0 | 64.3 | 0 | Supplements | Lung cancer (n=103);  Prostate cancer (n=1008)  Colorectal cancer (n=162) | 14641 | Lung cancer 0.95 (0.64, 1.39)  Prostate cancer 1.02 (0.90, 1.15)  Colorectal cancer 0.86 (0.63, 1.17) | Age, PHS cohort (original PHS I participant, new PHS participant), and randomized treatment assignment (beta carotene, multivitamin, and either vitamin E or vitamin C) and stratified on baseline cancer. |

JPHC: Japan Public Health Center-based Prospective Study; NCSDC: The Netherlands Cohort Study on Diet and Cancer; LWS: Leisure World Study; VITAL: VITamins And Lifestyle study; SCHS: Singapore Chinese Health Study; NHNES: National Health and Nutrition Examination Survey Epidemiologic Follow-up Study; EPIC: European Prospective Investigation into Cancer and Nutrition; I-EPIC: Italian EPIC; NHS: Nurses' Health Study; NCS: Netherlands Cohort Study; IWHS: Iowa women's health Study; SMSC: Swedish Mammography Screening Cohort; WHI: WHI observational study; NBSS: Canadian National Breast Screening Study; E3N: Etude Epidémiologique auprès de femmes de la Mutuelle Générale de l'Education Nationale (E3N) prospective cohort; NHSII: Nurses' Health Study II; PLCO: Prostate, Lung, Colorectal, and Ovarian Cancer Screening Trial; DPCS: Danish Prospective Cohort Study; WES: Western Electric Study; NIH-AARP: NIH-AARP Diet and Health Study; SWHS: Shanghai Women's Health Study; SMHS: Shanghai Men's Health Study; PHSII: the Physicians’ Health Study II. UK-DCC: UK Dietary Cohort Consortium. WACS: Women's Antioxidant Cardiovascular Study

**Additional file 3 -** Figure S1. Flow diagrams of the literature research.


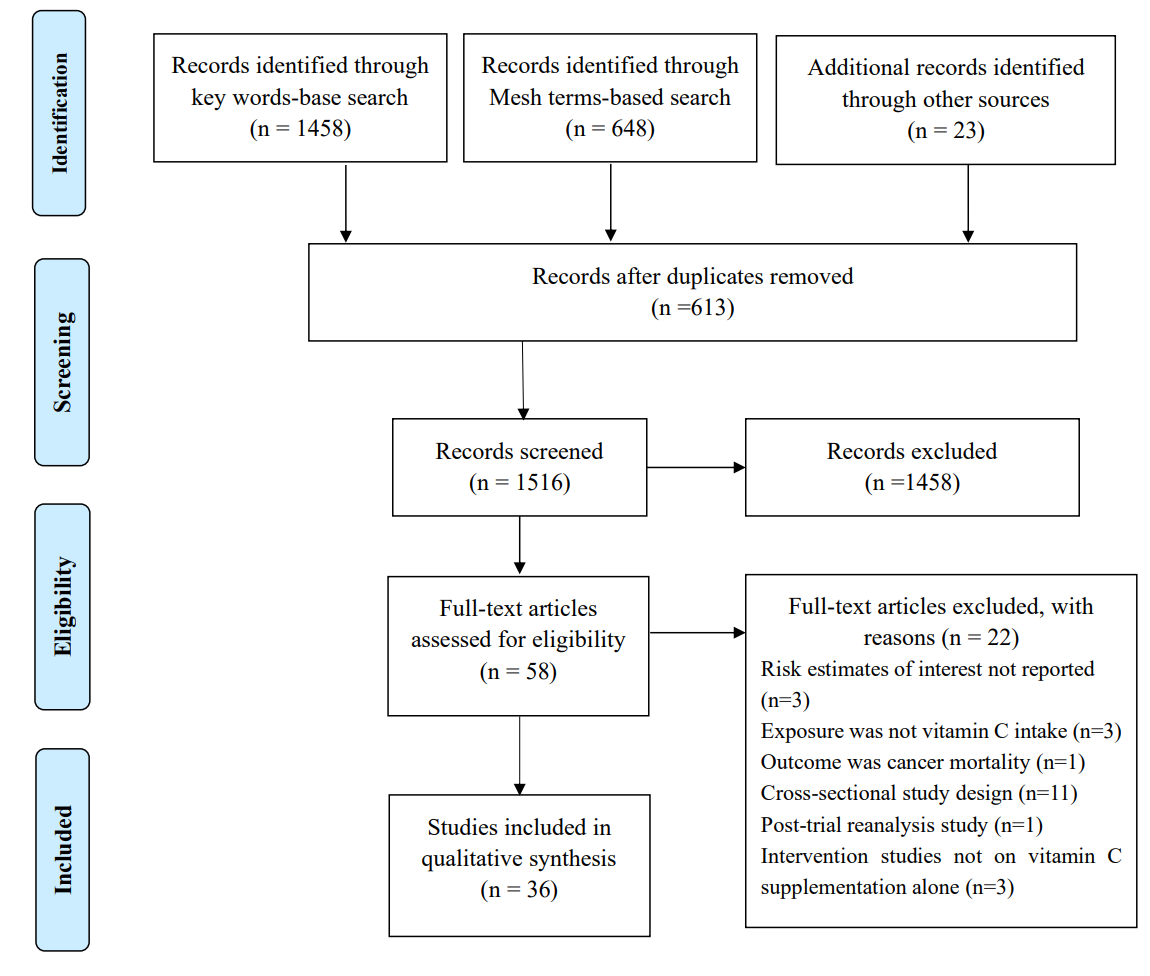


**Additional file 3 –**Figure **S2**.  **The association of dietary and supplemental vitamin C intakes with lung cancer**

**
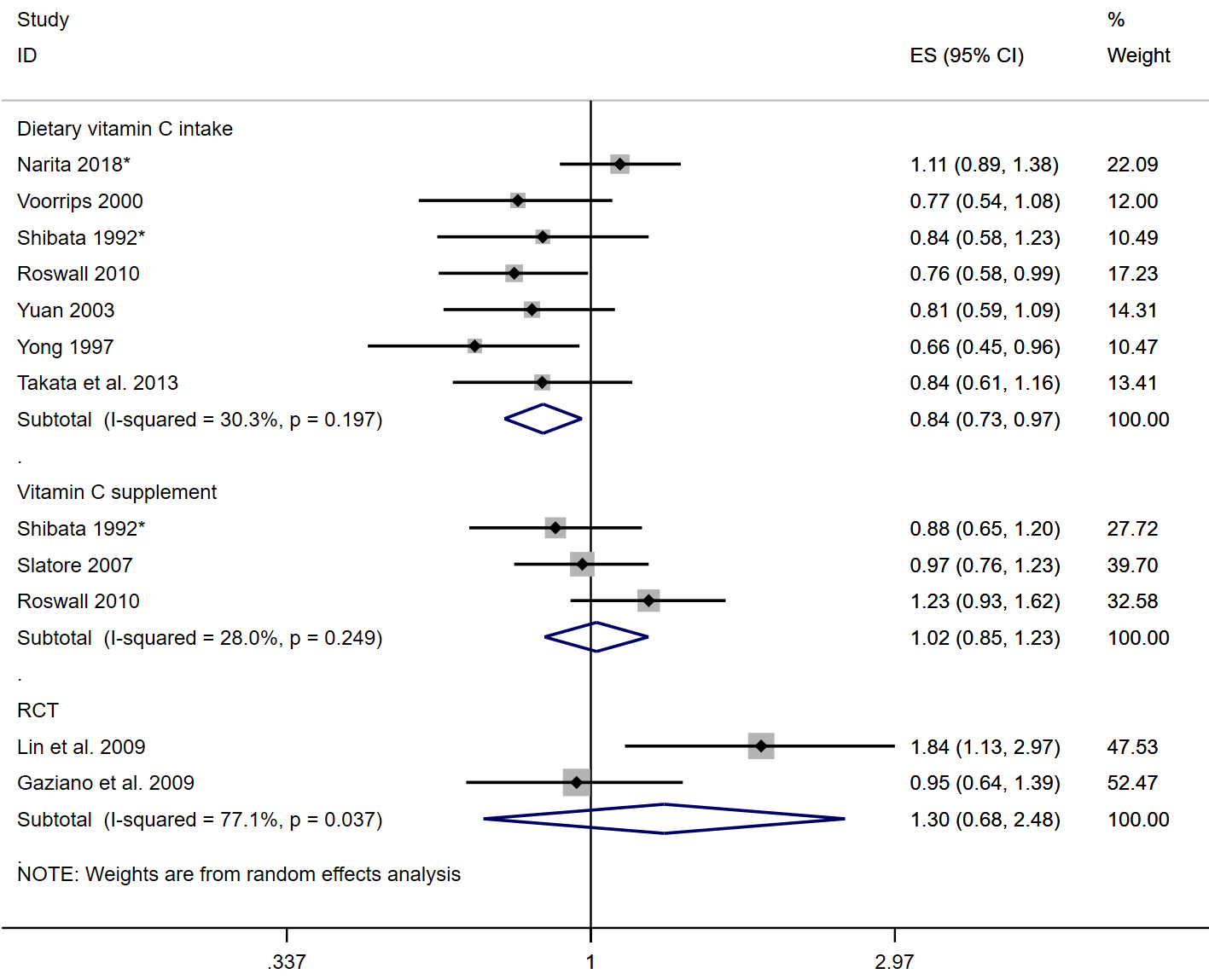
**

**Additional file 3 –**Figure **S3**. **The association of dietary, supplemental and total vitamin C intakes with breast cancer**

**
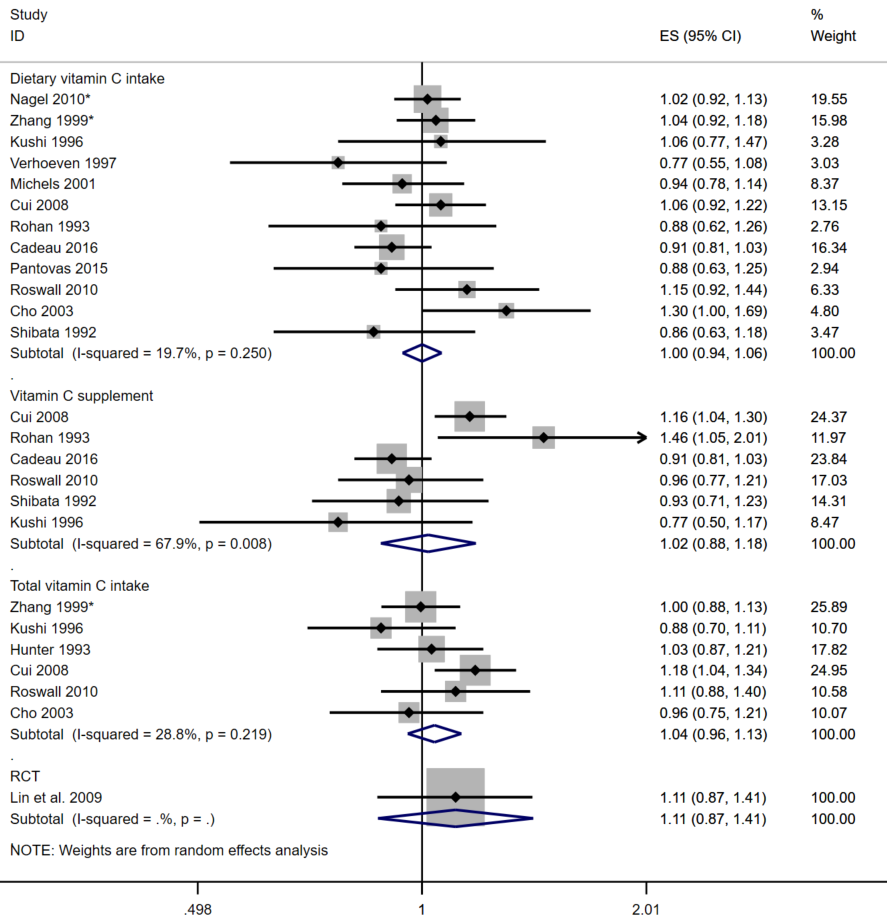
**

**Additional file 3 –** Figure S4.  **The association of dietary, supplemental and total vitamin C intakes with incident prostate cancer**

**
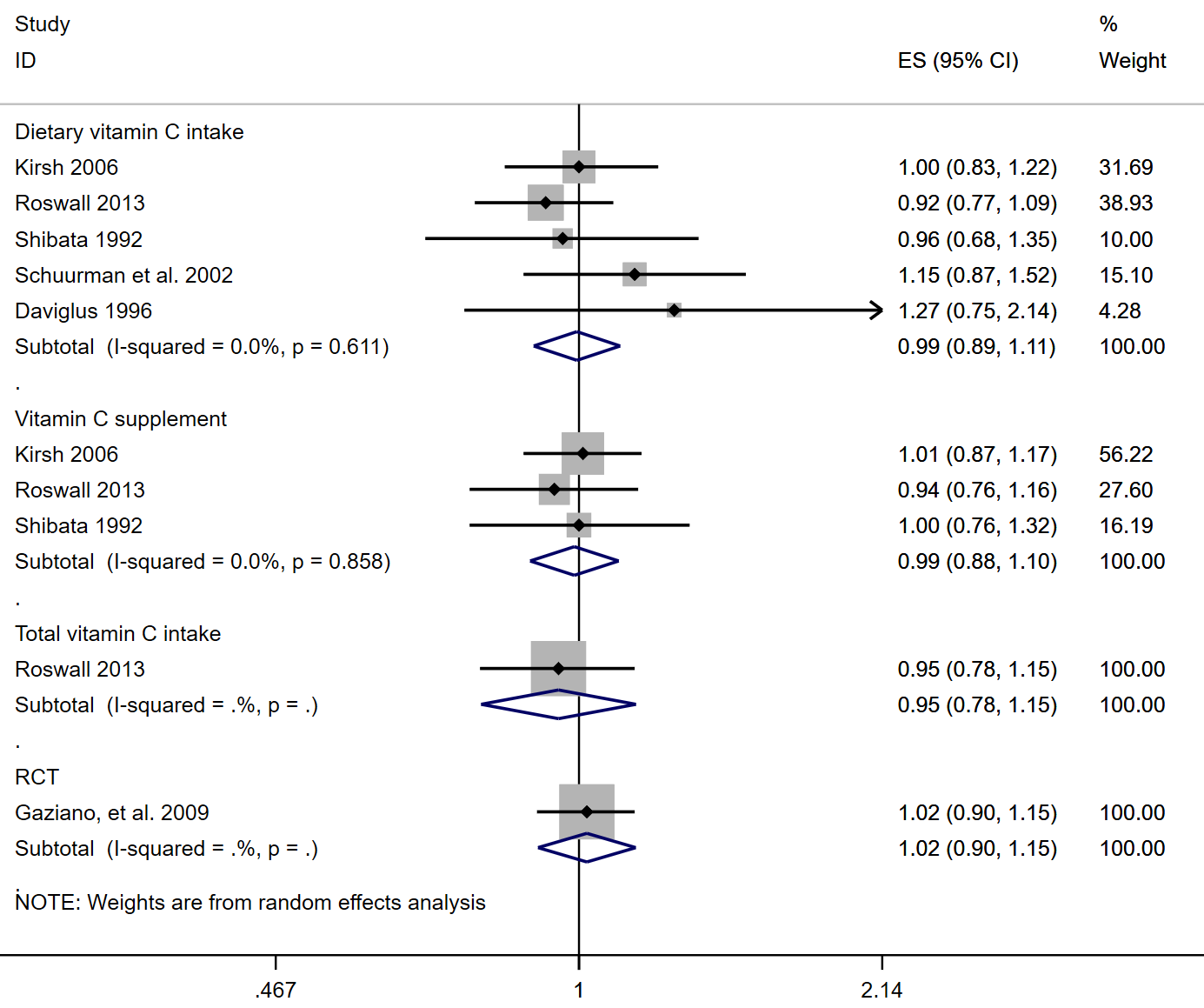
**

**Additional file 3 –** Figure S5.  **The association of dietary vitamin C intakes with incident colorectal cancer**

**
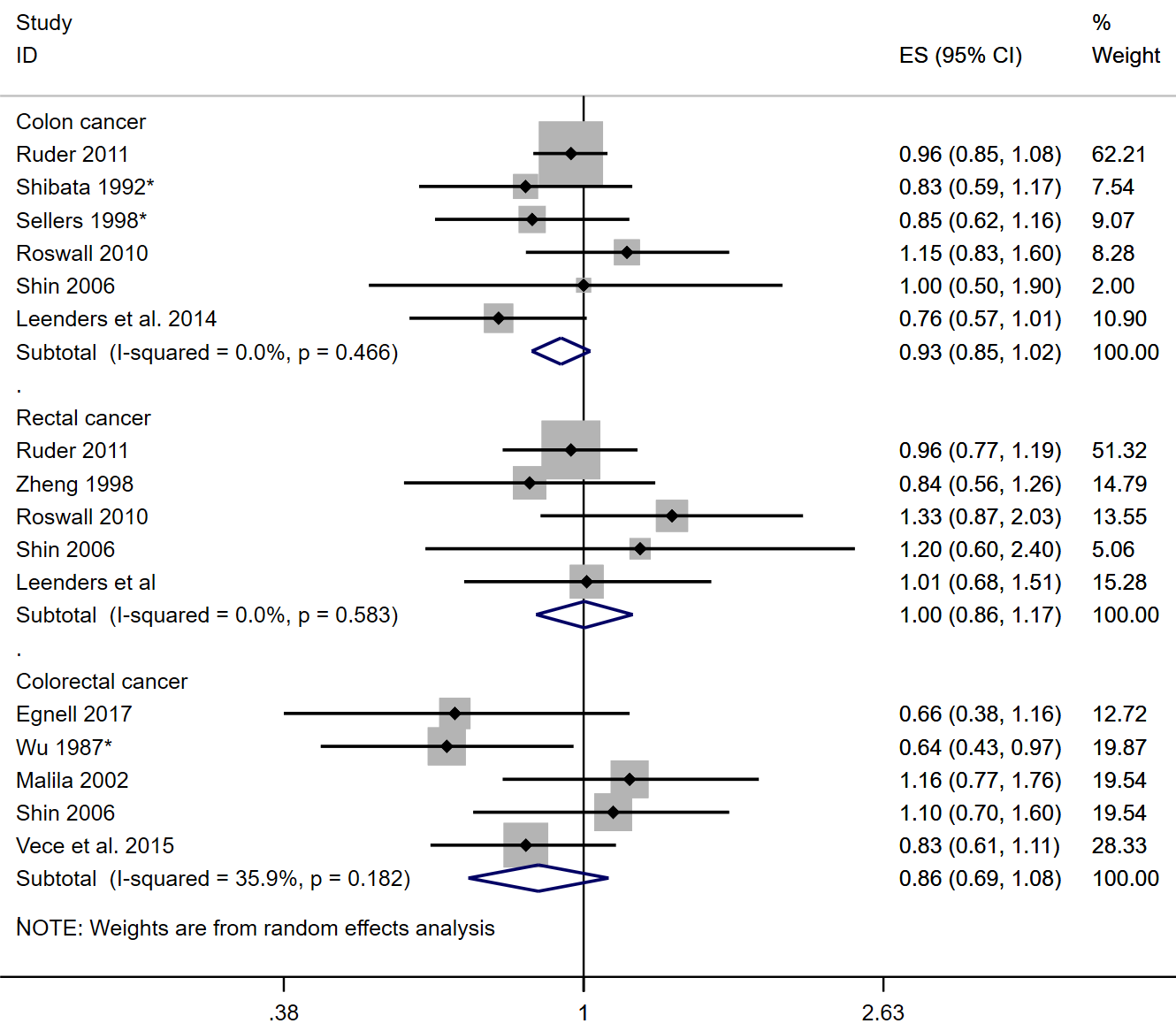
**

**Additional file 3 –** Figure S6.  **The association of supplemental vitamin C intakes with incident colorectal cancer**

**
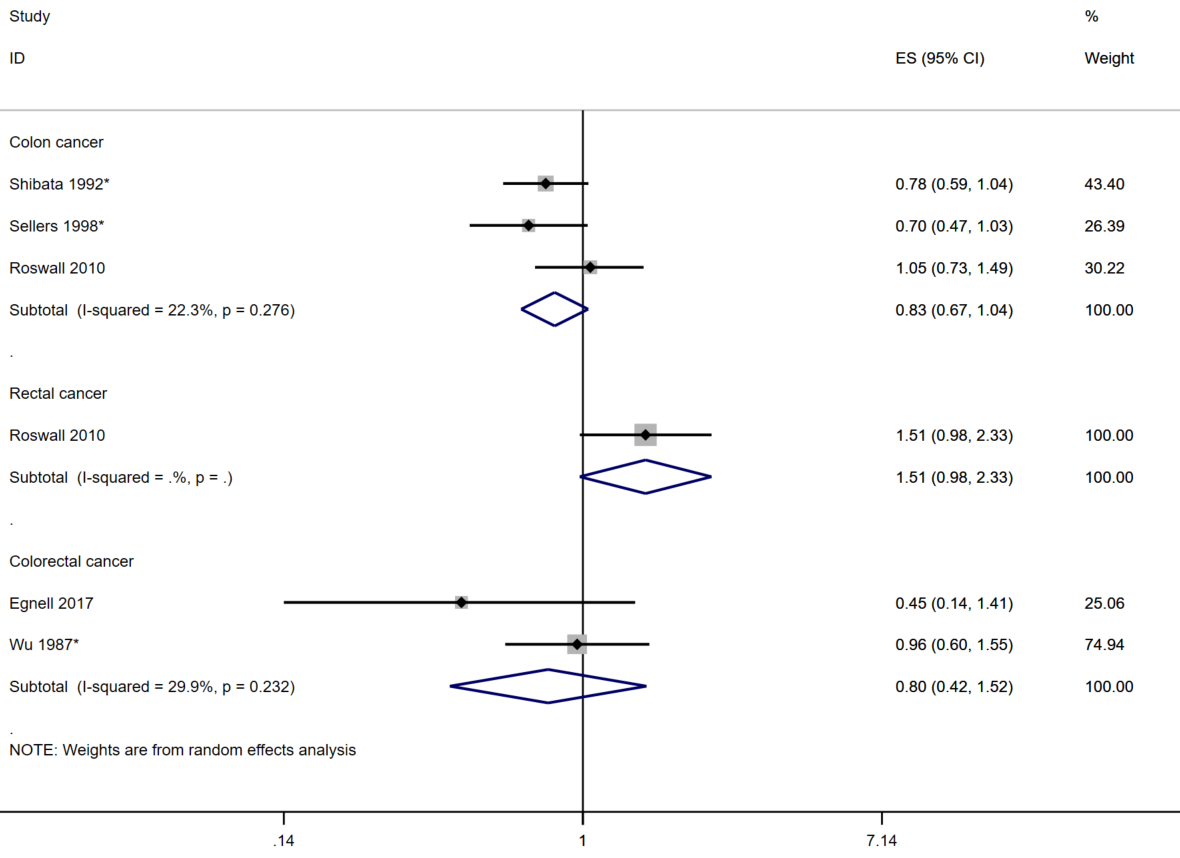
**

**Additional file 3 –** Figure S7. **The association of total vitamin C intakes with incident colorectal cancer**

**
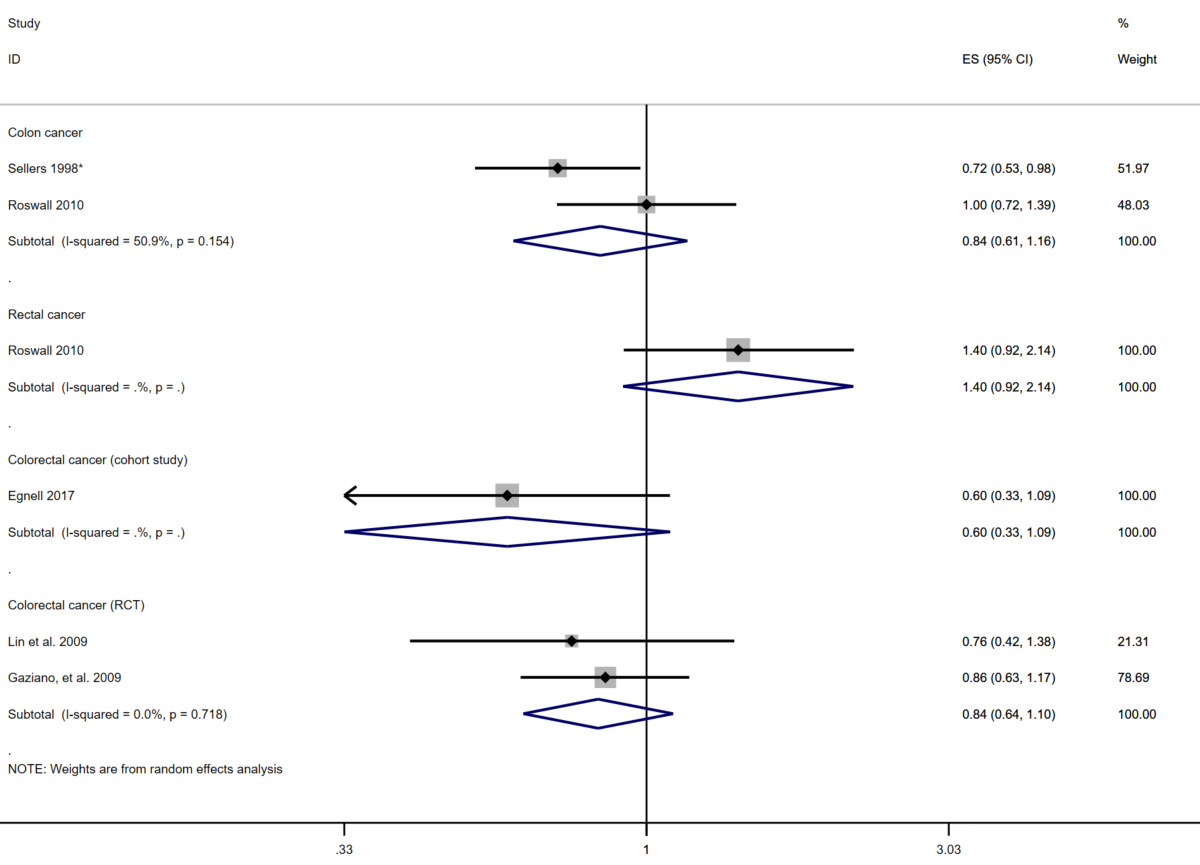
**

**Additional file 3 –**Figure **S8**.  **Publication bias among the included cohort studies**

**Lung cancer (a)**

**Dietary vitamin C and lung cancer (Egger’s test: p=0.038) Supplemental vitamin C intake and lung cancer (Egger’s test: p=0.930)**

**Breast cancer (b)**

**Dietary vitamin C intake and breast cancer (Egger’s test: p=0.634) Supplemental vitamin C intake and breast cancer (Egger’s test: p=0.832)**

**Breast cancer (b)**

**Total vitamin C intake and Breast cancer (Egger’s test: p=0.389)**

**Prostate cancer (c)**

**Dietary vitamin C intake and prostate cancer (Egger’s test: p=0.479) Supplemental vitamin C intake and prostate cancer (Egger’s test: p=0.718)**

**Colorectal/colon/rectal cancer (d)**

**Dietary vitamin C intake and Colorectal (or colon/rectal) cancer, (Egger’s test: p=0.855 for CRC; p=0.659 for colon cancer; p=0.456 for rectal cancer; Overall p=0.877)**

**Colorectal/colon/rectal cancer (d)**

**Supplemental vitamin C intake and colorectal or colon cancer (egg’s test: p value is not available for rectal cancer or colorectal cancer due to limited publications; p=0.934 for colon cancer; Overall p=0.894)**

**Colorectal/colon/rectal cancer (d)**

**Total vitamin C intake and colorectal (or colon/rectal cancer) (egg’s test: p value is not available for individual colon or rectal cancer due to limited publications, p=0.344 for colorectal cancer; overall p value was 0.855)**

**References**

1. Zheng JS, Luan J, Sofianopoulou E, et al. Plasma Vitamin C and Type 2 Diabetes: Genome-Wide Association Study and Mendelian Randomization Analysis in European Populations. *Diabetes Care* 2020
2. Genomes Project C, Auton A, Brooks LD, et al. A global reference for human genetic variation. *Nature* 2015;526(7571):68-74.
3. Yang J, Lee SH, Goddard ME, et al. GCTA: a tool for genome-wide complex trait analysis. *Am J Hum Genet* 2011;88(1):76-82.
4. Sudlow C, Gallacher J, Allen N, et al. UK biobank: an open access resource for identifying the causes of a wide range of complex diseases of middle and old age. *PLoS Med* 2015;12(3):e1001779.
5. Wang Y, McKay JD, Rafnar T, et al. Rare variants of large effect in BRCA2 and CHEK2 affect risk of lung cancer. *Nat Genet* 2014;46(7):736-41.
6. Hemani G, Zheng J, Elsworth B, et al. The MR-Base platform supports systematic causal inference across the human phenome. *Elife* 2018;7
7. Schumacher FR, Al Olama AA, Berndt SI, et al. Association analyses of more than 140,000 men identify 63 new prostate cancer susceptibility loci. *Nat Genet* 2018;50(7):928-36.
8. Zhang H, Ahearn TU, Lecarpentier J, et al. Genome-wide association study identifies 32 novel breast cancer susceptibility loci from overall and subtype-specific analyses. *Nat Genet* 2020;52(6):572-81.
9. Milne RL, Kuchenbaecker KB, Michailidou K, et al. Identification of ten variants associated with risk of estrogen-receptor-negative breast cancer. *Nat Genet* 2017;49(12):1767-78.
10. McKay JD, Hung RJ, Han Y, et al. Large-scale association analysis identifies new lung cancer susceptibility loci and heterogeneity in genetic susceptibility across histological subtypes. *Nat Genet* 2017;49(7):1126-32.
11. Ahearn TU, Zhang H, Michailidou K, et al. Common breast cancer risk loci predispose to distinct tumor subtypes. *bioRxiv* 2020
12. Law PJ, Timofeeva M, Fernandez-Rozadilla C, et al. Association analyses identify 31 new risk loci for colorectal cancer susceptibility. *Nat Commun* 2019;10(1):2154.
13. Liu M, Jiang Y, Wedow R, et al. Association studies of up to 1.2 million individuals yield new insights into the genetic etiology of tobacco and alcohol use. *Nat Genet* 2019;51(2):237-44.
14. Verbanck M, Chen CY, Neale B, et al. Detection of widespread horizontal pleiotropy in causal relationships inferred from Mendelian randomization between complex traits and diseases. *Nat Genet* 2018;50(5):693-98.
15. Burgess S, Thompson SG. Interpreting findings from Mendelian randomization using the MR-Egger method. *Eur J Epidemiol* 2017;32(5):377-89.
16. Stephen Burgess JB, Frank Dudbridge, Simon G Thompson. Robust instrumental variable methods using multiple candidate instruments with application to Mendelian randomization. *arXiv:160603729v2* 2018
17. Qingyuan Zhao JW, Gibran Hemani, Jack Bowden, Dylan S. Small. Statistical inference in two-sample summary-data Mendelian randomization using robust adjusted profile score. *arXiv:180109652v3* 2019
18. Bowden J, Davey Smith G, Haycock PC, et al. Consistent Estimation in Mendelian Randomization with Some Invalid Instruments Using a Weighted Median Estimator. *Genet Epidemiol* 2016;40(4):304-14.
19. Hartwig FP, Davey Smith G, Bowden J. Robust inference in summary data Mendelian randomization via the zero modal pleiotropy assumption. *Int J Epidemiol* 2017;46(6):1985-98.
20. Narita S, Saito E, Sawada N, et al. JPHC Study Group. Dietary consumption of antioxidant vitamins and subsequent lung cancer risk: The Japan Public Health Center-based prospective study. *Int J Cancer.* 2018; 142(12):2441-2460.
21. Voorrips LE, Goldbohm RA, Brants HA, et al. A prospective cohort study on antioxidant and folate intake and male lung cancer risk. *Cancer Epidemiol Biomarkers Prev.* 2000; 9(4):357-65.
22. Shibata A, Paganini-Hill A, Ross RK, Henderson BE. Intake of vegetables, fruits, beta-carotene, vitamin C and vitamin supplements and cancer incidence among the elderly: a prospective study. *Br J Cancer.* 1992; 66(4):673-9.
23. Slatore CG, Littman AJ, Au DH, Satia JA, White E. Long-term use of supplemental multivitamins, vitamin C, vitamin E, and folate does not reduce the risk of lung cancer. *Am J Respir Crit Care Med.* 2008; 177(5):524-30.
24. Roswall N, Olsen A, Christensen J, Dragsted LO, Overvad K, Tjønneland A. Source-specific effects of micronutrients in lung cancer prevention. *Lung Cancer.* 2010 Mar;67(3):275-81.
25. Yuan JM, Stram DO, Arakawa K, Lee HP, Yu MC. Dietary cryptoxanthin and reduced risk of lung cancer: the Singapore Chinese Health Study. Cancer Epidemiol Biomarkers Prev. 2003; 12(9):890-8.
26. Yong LC, Brown CC, Schatzkin A, Dresser CM, Slesinski MJ, Cox CS, Taylor PR. Intake of vitamins E, C, and A and risk of lung cancer. The NHANES I epidemiologic followup study. First National Health and Nutrition Examination Survey. *Am J Epidemiol.* 1997;146(3):231-43.
27. Nagel G, Linseisen J, van Gils CH, et al. Dietary beta-carotene, vitamin C and E intake and breast cancer risk in the European Prospective Investigation into Cancer and Nutrition (EPIC). Breast Cancer Res Treat. 2010; 119(3):753-65.
28. Zhang S, Hunter DJ, Forman MR, et al. Dietary carotenoids and vitamins A, C, and E and risk of breast cancer. *J Natl Cancer Inst.* 1999; 91(6):547-56.
29. Verhoeven DT, Assen N, Goldbohm RA, et al. Vitamins C and E, retinol, beta-carotene and dietary fibre in relation to breast cancer risk: a prospective cohort study. *Br J Cancer.* 1997;75(1):149-55.
30. Kushi LH, Fee RM, Sellers TA, Zheng W, Folsom AR. Intake of vitamins A, C, and E and postmenopausal breast cancer. The Iowa Women's Health Study. *Am J Epidemiol.* 1996;144(2):165-74.
31. Hunter DJ, Manson JE, Colditz GA, et al. A prospective study of the intake of vitamins C, E, and A and the risk of breast cancer. *N Engl J Med.* 1993; 329(4):234-40.
32. Michels KB, Holmberg L, Bergkvist L, Ljung H, Bruce A, Wolk A. Dietary antioxidant vitamins, retinol, and breast cancer incidence in a cohort of Swedish women. *Int J Cancer*. 2001; 91(4):563-7.
33. Cui Y, Shikany JM, Liu S, Shagufta Y, Rohan TE. Selected antioxidants and risk of hormone receptor-defined invasive breast cancers among postmenopausal women in the Women's Health Initiative Observational Study. *Am J Clin Nutr.* 2008; 87(4):1009-18.
34. Rohan TE, Howe GR, Friedenreich CM, Jain M, Miller AB. Dietary fiber, vitamins A, C, and E, and risk of breast cancer: a cohort study. *Cancer Causes Control.* 1993; 4(1):29-37.
35. Cadeau C, Fournier A, Mesrine S, Clavel-Chapelon F, Fagherazzi G, Boutron-Ruault MC. Vitamin C supplement intake and postmenopausal breast cancer risk: interaction with dietary vitamin C. *Am J Clin Nutr.* 2016; 104(1):228-34.
36. Pantavos A, Ruiter R, Feskens EF, de Keyser CE, Hofman A, Stricker BH, Franco OH, Kiefte-de Jong JC. Total dietary antioxidant capacity, individual antioxidant intake and breast cancer risk: the Rotterdam Study. *Int J Cancer.* 2015;136(9):2178-86.
37. Roswall N, Olsen A, Christensen J, Dragsted LO, Overvad K, Tjønneland A. Micronutrient intake and breast cancer characteristics among postmenopausal women. *Eur J Cancer Prev.* 2010; 19(5):360-5.
38. Cho E, Spiegelman D, Hunter DJ, et al. Premenopausal intakes of vitamins A, C, and E, folate, and carotenoids, and risk of breast cancer. *Cancer Epidemiol Biomarkers Prev.* 2003;12(8):713-20.
39. Kirsh VA, Hayes RB, Mayne ST, et al. Supplemental and dietary vitamin E, beta-carotene, and vitamin C intakes and prostate cancer risk. J Natl Cancer Inst. 2006; 98(4):245-54.
40. Roswall N, Larsen SB, Friis S, Outzen M, Olsen A, Christensen J, Dragsted LO, Tjønneland A. Micronutrient intake and risk of prostate cancer in a cohort of middle-aged, Danish men. *Cancer Causes Control.* 2013; 24(6):1129-35.
41. Daviglus ML, Dyer AR, Persky V,et al. Dietary beta-carotene, vitamin C, and risk of prostate cancer: results from the Western Electric Study. *Epidemiology.* 1996; 7(5):472-7.
42. Ruder EH, Thiébaut AC, Thompson FE,et al. Adolescent and mid-life diet: risk of colorectal cancer in the NIH-AARP Diet and Health Study. *Am J Clin Nutr.* 2011 Dec;94(6):1607-19.
43. Egnell M, Fassier P, Lécuyer L, et al. Antioxidant intake from diet and supplements and risk of digestive cancers in middle-aged adults: results from the prospective NutriNet-Santé cohort. *Br J Nutr.* 2017; 118(7):541-549.
44. Wu AH, Paganini-Hill A, Ross RK, Henderson BE. Alcohol, physical activity and other risk factors for colorectal cancer: a prospective study. *Br J Cancer.* 1987; 55(6):687-94.
45. Sellers TA, Bazyk AE, Bostick RM, Kushi LH, Olson JE, Anderson KE, Lazovich D, Folsom AR. Diet and risk of colon cancer in a large prospective study of older women: an analysis stratified on family history (Iowa, United States). *Cancer Causes Control.* 1998 Aug;9(4):357-67.
46. Zheng W, Anderson KE, Kushi LH, Sellers TA, Greenstein J, Hong CP, Cerhan JR, Bostick RM, Folsom AR. A prospective cohort study of intake of calcium, vitamin D, and other micronutrients in relation to incidence of rectal cancer among postmenopausal women. Cancer Epidemiol Biomarkers Prev. 1998 Mar;7(3):221-5.
47. Malila N, Virtamo J, Virtanen M, Pietinen P, Albanes D, Teppo L. Dietary and serum alpha-tocopherol, beta-carotene and retinol, and risk for colorectal cancer in male smokers. Eur J Clin Nutr. 2002 Jul;56(7):615-21. doi: 10.1038/sj.ejcn.1601366.
48. Roswall N, Olsen A, Christensen J, Dragsted LO, Overvad K, Tjønneland A. Micronutrient intake and risk of colon and rectal cancer in a Danish cohort. Cancer Epidemiol. 2010 Feb;34(1):40-6.
49. Vece MM, Agnoli C, Grioni S, Sieri S, Pala V, Pellegrini N, Frasca G, Tumino R, Mattiello A, Panico S, Bendinelli B, Masala G, Ricceri F, Sacerdote C, Krogh V. Dietary Total Antioxidant Capacity and Colorectal Cancer in the Italian EPIC Cohort. PLoS One. 2015 Nov 13;10(11):e0142995.
50. Shin A, Li H, Shu XO, Yang G, Gao YT, Zheng W. Dietary intake of calcium, fiber and other micronutrients in relation to colorectal cancer risk: Results from the Shanghai Women's Health Study. Int J Cancer. 2006 Dec 15;119(12):2938-42.
51. Leenders M, Leufkens AM, Siersema PD, van Duijnhoven FJ, Vrieling A, Hulshof PJ, van Gils CH, Overvad K, Roswall N, Kyrø C, Boutron-Ruault MC, Fagerhazzi G, Cadeau C, Kühn T, Johnson T, Boeing H, Aleksandrova K, Trichopoulou A, Klinaki E, Androulidaki A, Palli D, Grioni S, Sacerdote C, Tumino R, Panico S, Bakker MF, Skeie G, Weiderpass E, Jakszyn P, Barricarte A, María Huerta J, Molina-Montes E, Argüelles M, Johansson I, Ljuslinder I, Key TJ, Bradbury KE, Khaw KT, Wareham NJ, Ferrari P, Duarte-Salles T, Jenab M, Gunter MJ, Vergnaud AC, Wark PA, Bueno-de-Mesquita HB. Plasma and dietary carotenoids and vitamins A, C and E and risk of colon and rectal cancer in the European Prospective Investigation into Cancer and Nutrition. Int J Cancer. 2014 Dec 15;135(12):2930-9.
52. Lin J, Cook NR, Albert C, Zaharris E, Gaziano JM, Van Denburgh M, Buring JE, Manson JE. Vitamins C and E and beta carotene supplementation and cancer risk: a randomized controlled trial. J Natl Cancer Inst. 2009 Jan 7;101(1):14-23.
53. Gaziano JM, Glynn RJ, Christen WG, Kurth T, Belanger C, MacFadyen J, Bubes V, Manson JE, Sesso HD, Buring JE. Vitamins E and C in the prevention of prostate and total cancer in men: the Physicians' Health Study II randomized controlled trial. JAMA. 2009 Jan 7;301(1):52-62.
54. Takata Y, Xiang YB, Yang G, Li H, Gao J, Cai H, Gao YT, Zheng W, Shu XO. Intakes of fruits, vegetables, and related vitamins and lung cancer risk: results from the Shanghai Men's Health Study (2002-2009). Nutr Cancer. 2013;65(1):51-61.
55. Schuurman AG, Goldbohm RA, Brants HA, van den Brandt PA. A prospective cohort study on intake of retinol, vitamins C and E, and carotenoids and prostate cancer risk (Netherlands). Cancer Causes Control. 2002 Aug;13(6):573-82.
