# Supplementary material for: Circulating vitamin C concentration and risk of cancers: a Mendelian randomization study
Source: BMC Med. 2021 Jul 30;19:171. doi: 10.1186/s12916-021-02041-1 (PMC8323227; doi:10.1186/s12916-021-02041-1)
Supplement: Supplementary file 4 — Additional file 4: Figure S1-S8. The forest and scatter plots for each SNP-CA association and the results of heterogeneity test. FigS1. Genetically predicted associations of plasma vitamin C with lung cancer in the UK biobank dataset. FigS2. Genetically predicted associations of plasma vitamin C with breast cancer in the UK biobank dataset. FigS3. Genetically predicted associations of plasma vitamin C with prostate cancer in the UK biobank dataset. FigS4. Genetically predicted associations of plasma vitamin C with colon cancer in the UK biobank dataset. FigS5. Genetically predicted associations of plasma vitamin C with rectal cancer in the UK biobank dataset. FigS6. Genetically predicted associations of plasma vitamin C with lung cancer in the dataset from International Lung Cancer Consortium (ILCCO). FigS7. Genetically predicted associations of plasma vitamin C with breast cancer in the dataset from the Breast Cancer Association Consortium (BCAC). FigS8. Genetically predicted associations of plasma vitamin C with prostate cancer in the dataset from the Prostate Cancer Association Group to Investigate Cancer Associated Alterations in the Genome (PRACTICAL). [file 12916_2021_2041_MOESM4_ESM.docx]

**Additional file 4**

**Additional file 4 – Figure 1: Genetically predicted associations of plasma vitamin C with lung cancer in the UK biobank dataset.** (A) Forest plot for associations of each vitamin C-related SNP with lung cancer risk. (B) Scatter plot of genetic association with the plasma vitamin C levels and genetic association with lung cancer. The results from the primary approach (random-effects inverse-variance-weighted method) were presented in the plots, while the results from other sensitivity analyses were presented in the tables below the plots.

1. **(B)**

| 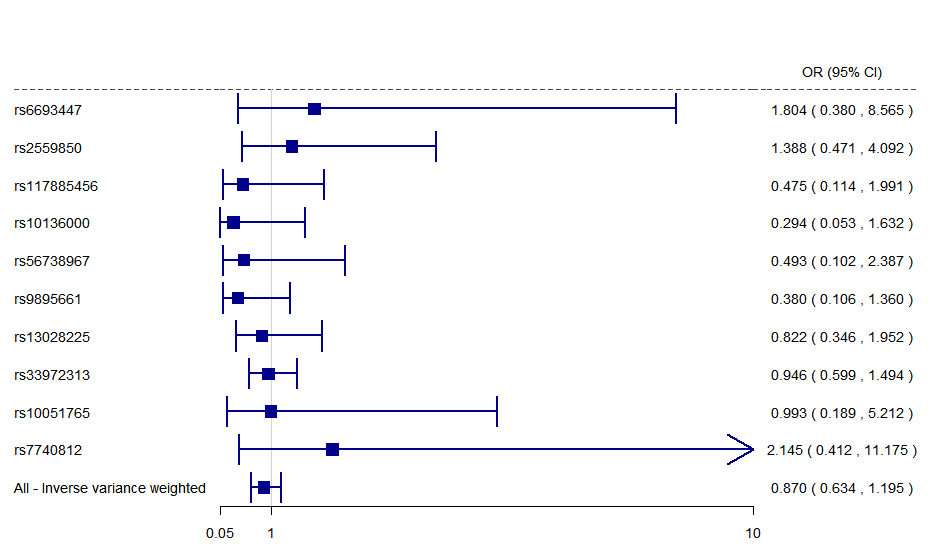 | 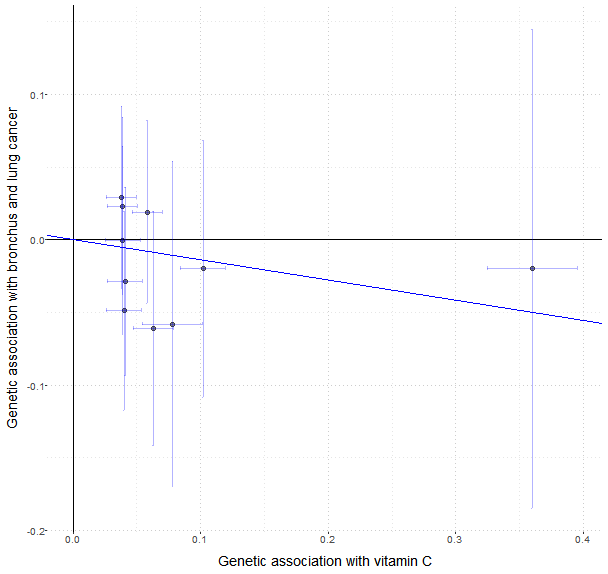 |
| --- | --- |

|  | OR | 95% CI | | p | Cochran’s Q test |
| --- | --- | --- | --- | --- | --- |
| Inverse-variance weighted | 0.870 | 0.634 | 1.195 | 0.390 | 0.614 |
| MR-Egger | 0.892 | 0.543 | 1.464 | 0.663 |  |
| Weighted median | 0.922 | 0.612 | 1.388 | 0.696 |  |
| MR-PRESSO | 0.870 | 0.655 | 1.156 | 0.362 |  |
| mode-based | 0.943 | 0.617 | 1.440 | 0.785 |  |
| MR-Robust | 0.875 | 0.712 | 1.075 | 0.202 |  |
| MR-RAPS | 0.869 | 0.625 | 1.208 | 0.404 |  |

**Additional file 4 – Figure 2:** **Genetically predicted associations of plasma vitamin C with breast cancer in the UK biobank dataset.** (A) Forest plot for associations of each vitamin C-related SNP with lung cancer risk. (B) Scatter plot of genetic association with the plasma vitamin C levels and genetic association with lung cancer. The results from the primary approach (random-effects inverse-variance-weighted method) were presented in the plots, while the results from other sensitivity analyses were presented in the tables below the plots.

1. **(B)**

| 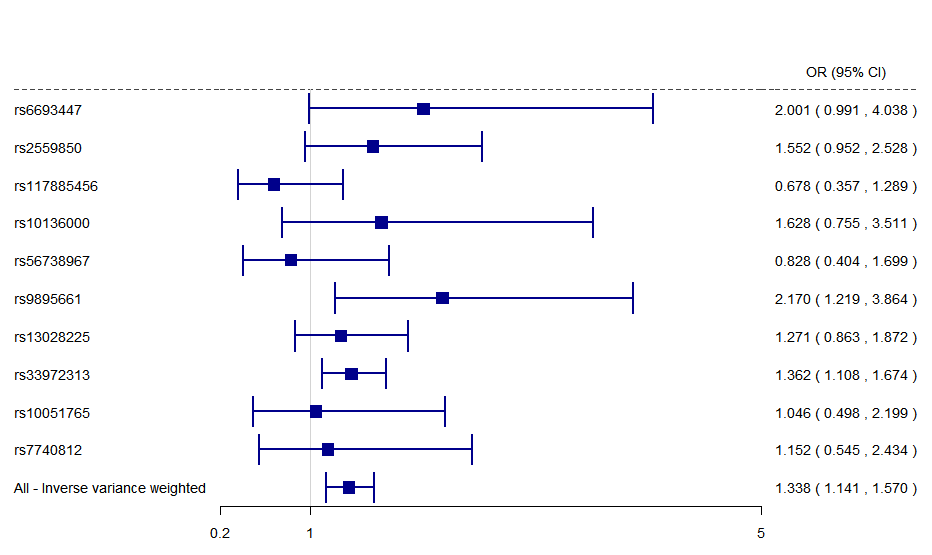 | 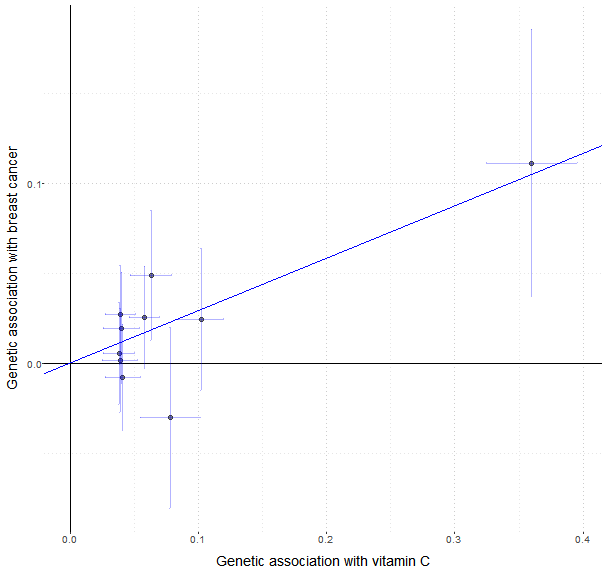 |
| --- | --- |

|  | OR | 95% CI | | p | Cochran’s Q test |
| --- | --- | --- | --- | --- | --- |
| Inverse-variance weighted | 1.338 | 1.141 | 1.570 | 0.000 | 0.259 |
| MR-Egger | 1.344 | 1.031 | 1.752 | 0.030 |  |
| Weighted median | 1.349 | 1.125 | 1.617 | 0.001 |  |
| MR-PRESSO | 1.338 | 1.141 | 1.570 | 0.006 |  |
| mode-based | 1.359 | 1.121 | 1.648 | 0.002 |  |
| MR-Robust | 1.344 | 1.214 | 1.489 | 0.000 |  |
| MR-RAPS | 1.351 | 1.153 | 1.583 | 0.000 |  |

**Additional file 4 – Figure 3:** **Genetically predicted associations of plasma vitamin C with prostate cancer in the UK biobank dataset.** (A) Forest plot for associations of each vitamin C-related SNP with lung cancer risk. (B) Scatter plot of genetic association with the plasma vitamin C levels and genetic association with lung cancer. The results from the primary approach (random-effects inverse-variance-weighted method) were presented in the plots, while the results from other sensitivity analyses were presented in the tables below the plots.

1. **(B)**

| 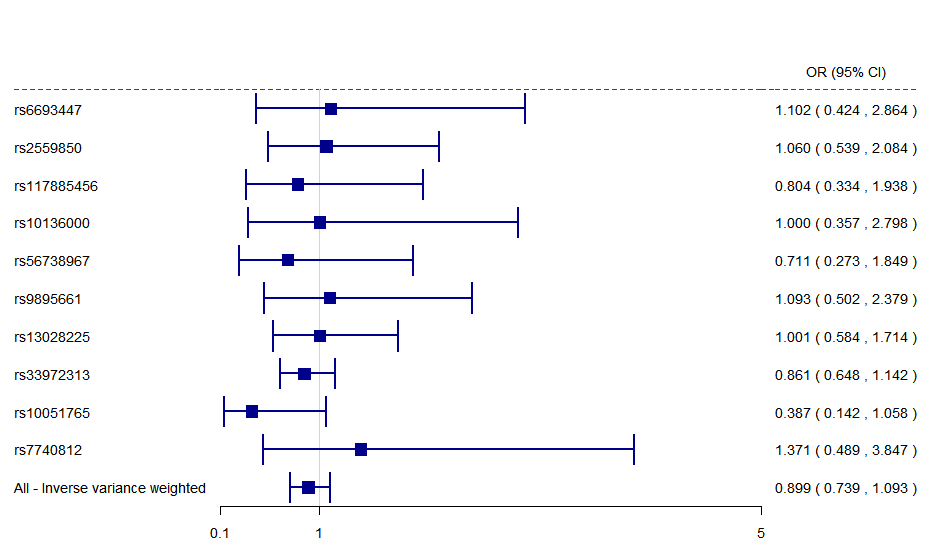 | 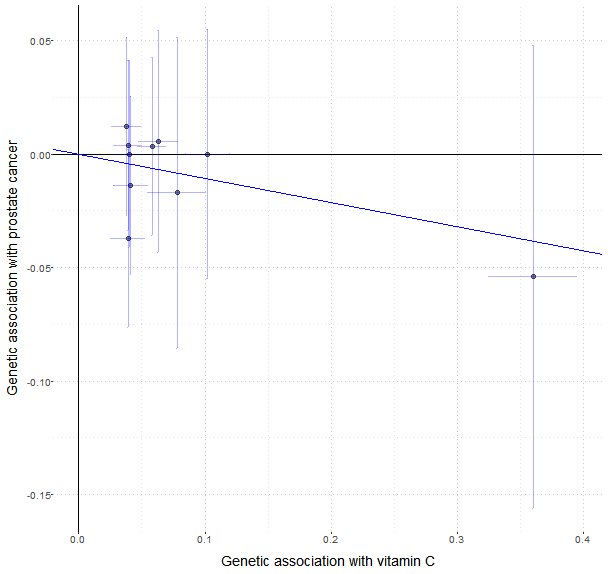 |
| --- | --- |

|  | OR | 95% CI | | p | Cochran’s Q test |
| --- | --- | --- | --- | --- | --- |
| Inverse-variance weighted | 0.899 | 0.739 | 1.093 | 0.286 | 0.243 |
| MR-Egger | 0.886 | 0.652 | 1.204 | 0.463 |  |
| Weighted median | 0.931 | 0.726 | 1.195 | 0.574 |  |
| MR-PRESSO | 0.899 | 0.782 | 1.033 | 0.168 |  |
| mode-based | 0.885 | 0.682 | 1.148 | 0.358 |  |
| MR-Robust | 0.913 | 0.752 | 1.109 | 0.360 |  |
| MR-RAPS | 0.904 | 0.737 | 1.108 | 0.331 |  |

**Additional file 4 – Figure 4:** **Genetically predicted associations of plasma vitamin C with colon cancer in the UK biobank dataset.** (A) Forest plot for associations of each vitamin C-related SNP with lung cancer risk. (B) Scatter plot of genetic association with the plasma vitamin C levels and genetic association with lung cancer. The results from the primary approach (random-effects inverse-variance-weighted method) were presented in the plots, while the results from other sensitivity analyses were presented in the tables below the plots.

**(A) (B)**

| 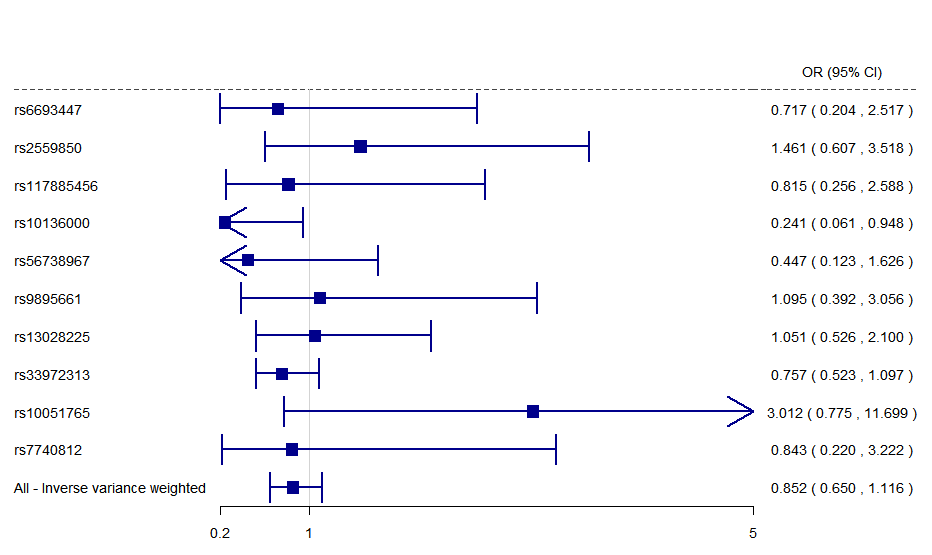 | 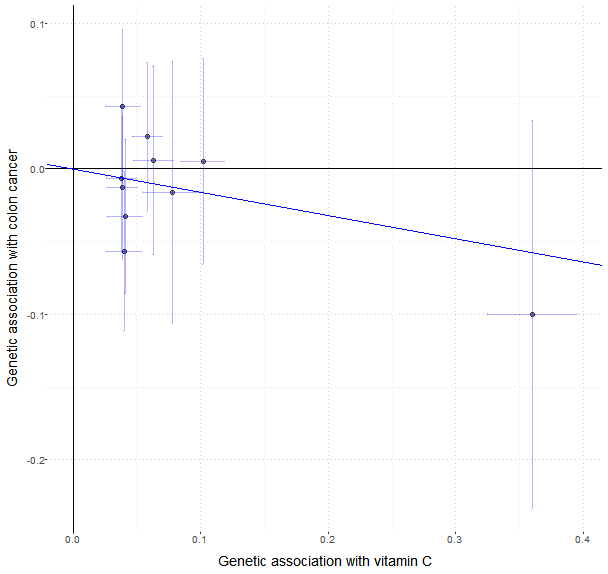 |
| --- | --- |

|  | OR | 95% CI | | p | Cochran’s Q test |
| --- | --- | --- | --- | --- | --- |
| Inverse-variance weighted | 0.852 | 0.650 | 1.116 | 0.245 | 0.347 |
| MR-Egger | 0.809 | 0.517 | 1.265 | 0.379 |  |
| Weighted median | 0.789 | 0.569 | 1.094 | 0.155 |  |
| MR-PRESSO | 0.852 | 0.650 | 1.116 | 0.275 |  |
| mode-based | 0.793 | 0.559 | 1.123 | 0.191 |  |
| MR-Robust | 0.849 | 0.687 | 1.048 | 0.128 |  |
| MR-RAPS | 0.849 | 0.649 | 1.110 | 0.231 |  |

**Additional file 4 – Figure 5:** **Genetically predicted associations of plasma vitamin C with rectal cancer in the UK biobank dataset.** (A) Forest plot for associations of each vitamin C-related SNP with lung cancer risk. (B) Scatter plot of genetic association with the plasma vitamin C levels and genetic association with lung cancer. The results from the primary approach (random-effects inverse-variance-weighted method) were presented in the plots, while the results from other sensitivity analyses were presented in the tables below the plots.

**(A) (B)**

| 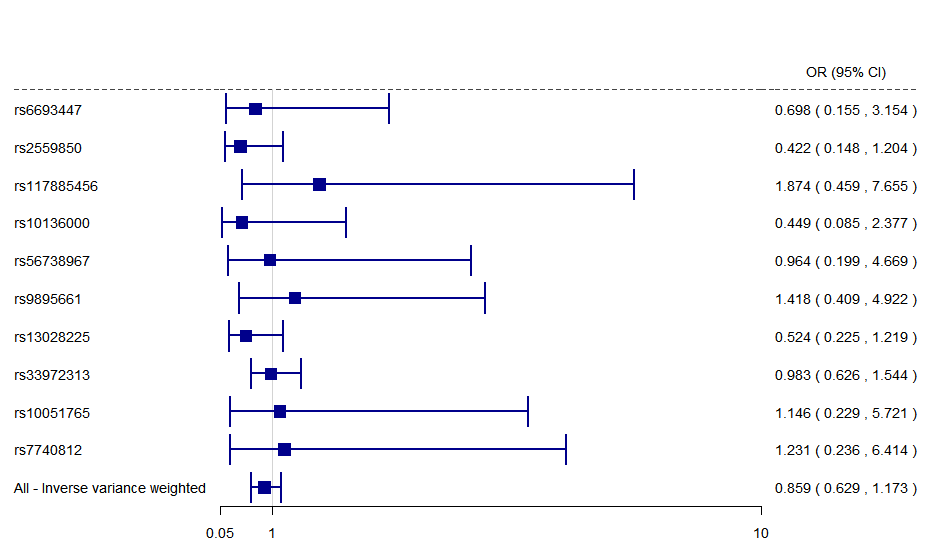 | 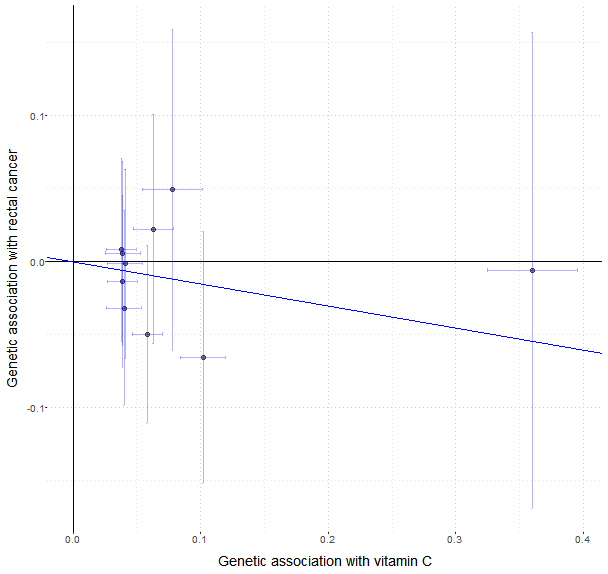 |
| --- | --- |

|  | OR | 95% CI | | p | Cochran’s Q test |
| --- | --- | --- | --- | --- | --- |
| Inverse-variance weighted | 0.859 | 0.629 | 1.173 | 0.340 | 0.719 |
| MR-Egger | 0.940 | 0.576 | 1.533 | 0.810 |  |
| Weighted median | 0.977 | 0.648 | 1.473 | 0.913 |  |
| MR-PRESSO | 0.859 | 0.663 | 1.113 | 0.280 |  |
| mode-based | 1.000 | 0.640 | 1.561 | 0.999 |  |
| MR-Robust | 0.874 | 0.633 | 1.206 | 0.412 |  |
| MR-RAPS | 0.858 | 0.621 | 1.187 | 0.355 |  |

**Additional file 4 – Figure 6:** **Genetically predicted associations of plasma vitamin C with lung cancer in the dataset from International Lung Cancer Consortium (ILCCO).** (A) Forest plot for associations of each vitamin C-related SNP with lung cancer risk. (B) Scatter plot of genetic association with the plasma vitamin C levels and genetic association with lung cancer. The results from the primary approach (random-effects inverse-variance-weighted method) were presented in the plots, while the results from other sensitivity analyses were presented in the tables below the plots.

**(A) (B)**

| 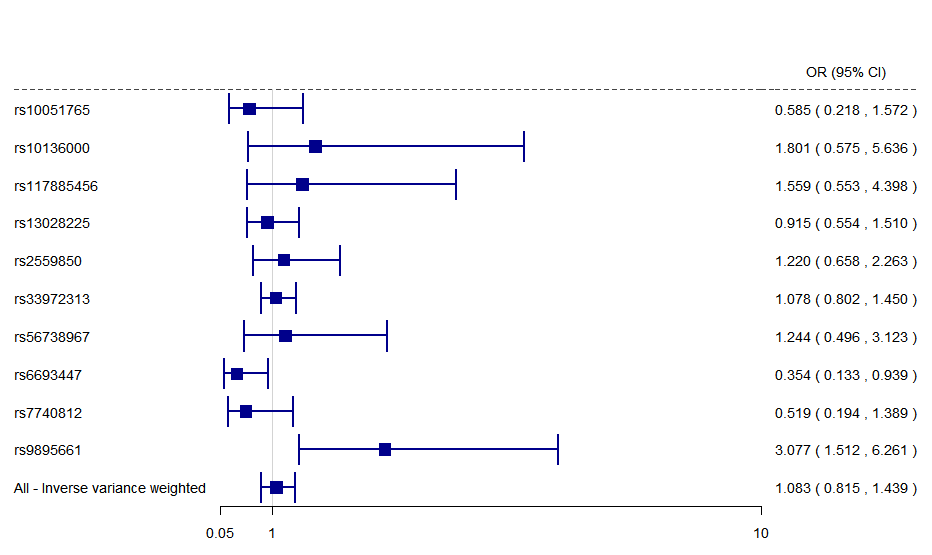 | 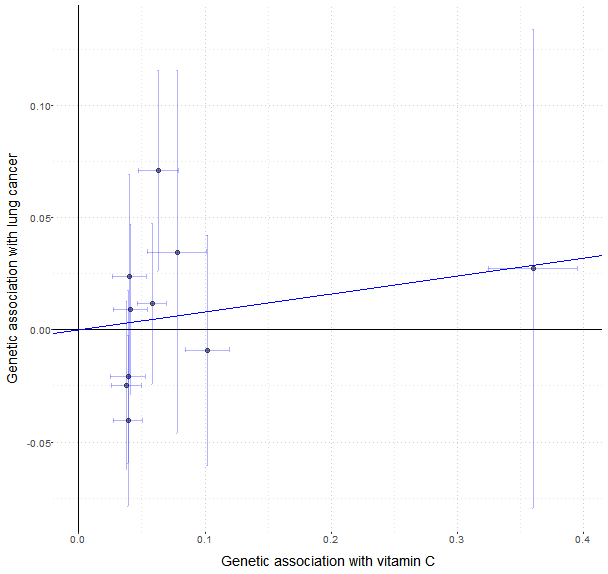 |
| --- | --- |

|  | OR | 95% CI | | p | Cochran’s Q test |
| --- | --- | --- | --- | --- | --- |
| Inverse-variance weighted | 1.083 | 0.815 | 1.439 | 0.584 | 0.026 |
| MR-Egger | 1.188 | 0.735 | 1.920 | 0.503 |  |
| Weighted median | 1.081 | 0.845 | 1.382 | 0.535 |  |
| MR-PRESSO | 1.083 | 0.815 | 1.439 | 0.598 |  |
| mode-based | 1.082 | 0.834 | 1.403 | 0.554 |  |
| MR-Robust | 1.066 | 0.891 | 1.276 | 0.483 |  |
| MR-RAPS | 1.069 | 0.845 | 1.352 |  |  |

**Additional file 4 – Figure 7:** **Genetically predicted associations of plasma vitamin C with breast cancer in the dataset from the Breast Cancer Association Consortium (BCAC).** (A) Forest plot for associations of each vitamin C-related SNP with lung cancer risk. (B) Scatter plot of genetic association with the plasma vitamin C levels and genetic association with lung cancer. The results from the primary approach (random-effects inverse-variance-weighted method) were presented in the plots, while the results from other sensitivity analyses were presented in the tables below the plots.

**(A) (B)**

| 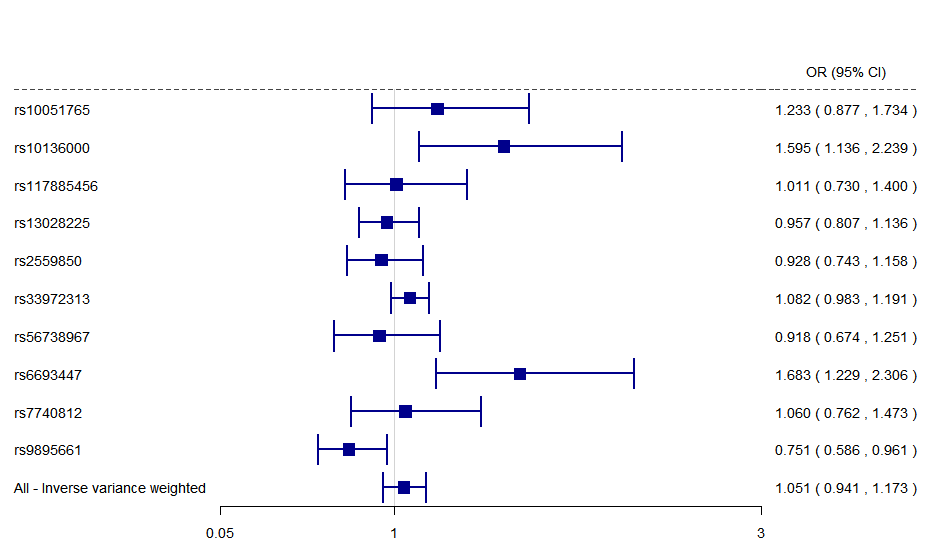 | 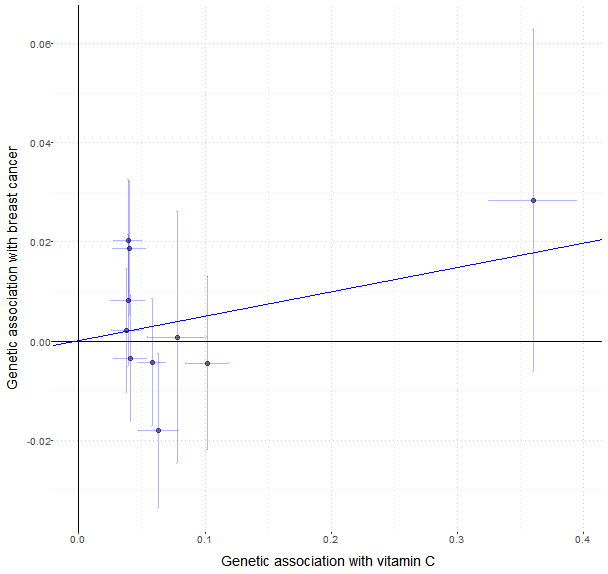 |
| --- | --- |

|  | OR | 95% CI | | p | Cochran’s Q test |
| --- | --- | --- | --- | --- | --- |
| Inverse-variance weighted | 1.051 | 0.941 | 1.173 | 0.378 | 0.002 |
| MR-Egger | 1.016 | 0.846 | 1.220 | 0.870 |  |
| Weighted median | 1.068 | 0.978 | 1.166 | 0.144 |  |
| MR-PRESSO | 1.055 | 0.970 | 1.146 | 0.250 |  |
| mode-based | 1.043 | 0.954 | 1.141 | 0.353 |  |
| MR-Robust | 1.048 | 0.966 | 1.138 | 0.256 |  |
| MR-RAPS | 1.054 | 0.968 | 1.147 | 0.223 |  |

**Additional file 4 – Figure 8:** **Genetically predicted associations of plasma vitamin C with prostate cancer in the dataset from the Prostate Cancer Association Group to Investigate Cancer Associated Alterations in the Genome (PRACTICAL).** (A) Forest plot for associations of each vitamin C-related SNP with lung cancer risk. (B) Scatter plot of genetic association with the plasma vitamin C levels and genetic association with lung cancer. The results from the primary approach (random-effects inverse-variance-weighted method) were presented in the plots, while the results from other sensitivity analyses were presented in the tables below the plots.

**(A) (B)**

| 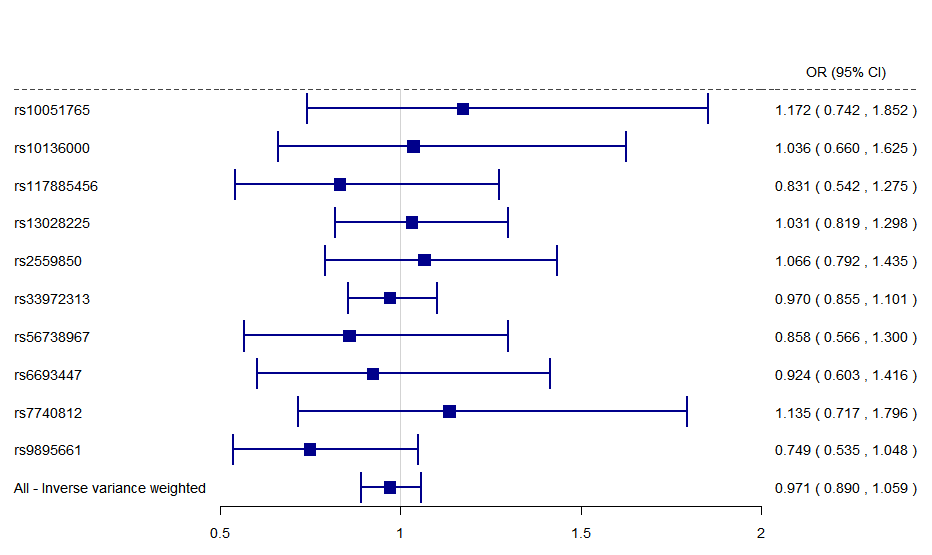 | 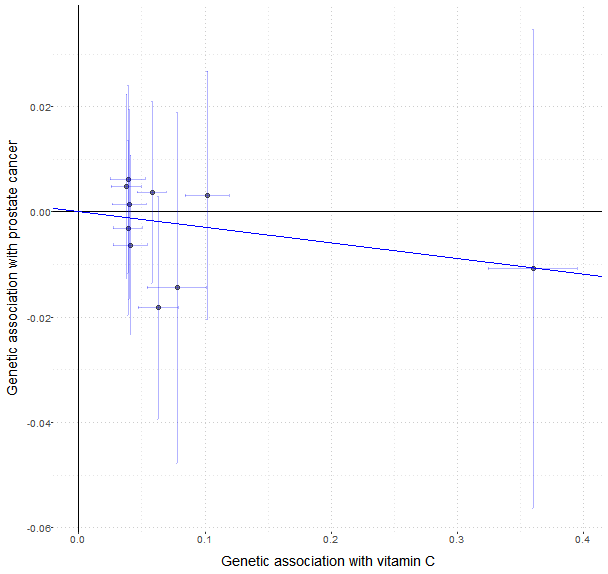 |
| --- | --- |

|  | OR | 95% CI | | p | Cochran’s Q test |
| --- | --- | --- | --- | --- | --- |
| Inverse-variance weighted | 0.971 | 0.890 | 1.059 | 0.502 | 0.834 |
| MR-Egger | 0.963 | 0.840 | 1.103 | 0.599 |  |
| Weighted median | 0.985 | 0.880 | 1.102 | 0.785 |  |
| MR-PRESSO | 0.971 | 0.910 | 1.036 | 0.391 |  |
| mode-based | 0.989 | 0.879 | 1.113 | 0.857 |  |
| MR-Robust | 0.975 | 0.922 | 1.030 | 0.368 |  |
| MR-RAPS | 0.973 | 0.889 | 1.064 | 0.545 |  |
